# Neutrophils drive endoplasmic reticulum stress-mediated apoptosis in cancer cells through arginase-1 release

**DOI:** 10.1038/s41598-021-91947-0

**Published:** 2021-06-15

**Authors:** Rósula García-Navas, Consuelo Gajate, Faustino Mollinedo

**Affiliations:** 1 Instituto de Biología Molecular y Celular del Cáncer, Centro de Investigación del Cáncer, Consejo Superior de Investigaciones Científicas (CSIC)-Universidad de Salamanca, Campus Miguel de Unamuno, 37007 Salamanca, Spain; 2 Centro de Investigación Biomédica en Red de Cáncer (CIBERONC), Salamanca, Spain; 3 Laboratory of Cell Death and Cancer Therapy, Department of Molecular Biomedicine, Centro de Investigaciones Biológicas Margarita Salas, Consejo Superior de Investigaciones Científicas (CSIC), C/Ramiro de Maeztu 9, 28040 Madrid, Spain

**Keywords:** Biochemistry, Cancer, Immunology, Oncology

## Abstract

Human neutrophils constitutively express high amounts of arginase-1, which depletes arginine from the surrounding medium and downregulates T-cell activation. Here, we have found that neutrophil arginase-1, released from activated human neutrophils or dead cells, induced apoptosis in cancer cells through an endoplasmic reticulum (ER) stress pathway. Silencing of *PERK* in cancer cells prevented the induction of ER stress and apoptosis. Arginase inhibitor Nω-hydroxy-nor-arginine inhibited apoptosis and ER stress response induced by conditioned medium from activated neutrophils. A number of tumor cell lines, derived from different tissues, were sensitive to neutrophil arginase-1, with pancreatic, breast, ovarian and lung cancer cells showing the highest sensitivity. Neutrophil-released arginase-1 and arginine deprivation potentiated the antitumor action against pancreatic cancer cells of the ER-targeted antitumor alkylphospholipid analog edelfosine. Our study demonstrates the involvement of neutrophil arginase-1 in cancer cell killing and highlights the importance and complex role of neutrophils in tumor surveillance and biology.

## Introduction

Polymorphonuclear neutrophils (PMNs) are the most abundant circulating blood leukocytes in humans and are an essential component of the innate immune system, providing the first line of cellular defense against infection and being the first responder to inflammation^1^. Neutrophils rapidly reach sites of infection and injury, releasing a variety of molecules to eliminate the infective agent and eliciting an acute inflammatory response. Neutrophils contain a complete armory of proteins able to degrade a wide variety of engulfed microorganisms, which can cause deleterious effects in neighboring tissues, following their extracellular release in uncontrolled inflammatory reactions. Most of these constituents are stored in three major neutrophil intracellular granules, namely gelatinase-rich tertiary, specific (secondary) and azurophilic (primary) granules, which play a critical role in neutrophil function through their mobilization by not yet fully defined mechanisms^2^. Neutrophils are emerging as double-edged swords in cancer, having been associated with both cancer progression and immunosurveillance against tumors, and therefore they can promote or inhibit cancer development^3–5^. An elevated neutrophil-to-lymphocyte ratio has become a prognostic indicator of poor survival in cancer^3,6^. Two arginase isozymes exist, arginase-1 and arginase-2, which differ in tissue distribution and intracellular localization^7^, and human neutrophils constitutively express high amounts of arginase-1^8^. Neutrophil arginase-1, released from neutrophils by sonication, depletes extracellular arginine and suppresses T-cell functions^9^. Arginine is one of the most versatile amino acids in animal cells, serving as precursor for the synthesis of proteins, polyamines, nitric oxide, urea, proline, glutamine, creatine and agmatine^10^. Polyamines are essential for cell proliferation and differentiation^11^, and increased polyamine synthesis seems to be necessary for neoplastic cell growth^12^. To support rapid growth with minimal energy expenditure, cancer cells reprogram their metabolism and favor import of arginine from exogenous sources, while downregulate energy-requiring arginine biosynthesis pathways^13^, thus rendering tumor cells particularly dependent on arginine for growth^14^. Treatment of cell cultures with arginase has been shown to reduce rapidly arginine in the medium to very low or negligible levels within minutes, and has been proven as effective as arginine-free medium^15^. Interestingly, cancer cells are more vulnerable to arginine depletion than normal counterparts^15,16^, and restriction of arginine availability has been advanced as a putative therapeutic approach in cancer treatment^14,17^. This cancer cell sensitivity to arginine deprivation is not determined by endogenous levels of arginine metabolic enzymes^18^.

In this study we show that human neutrophil arginase, corresponding to arginase-1, is able to induce apoptosis in a number of tumor cells derived from different tissues. Released neutrophil arginase depletes arginine in the extracellular medium, leading eventually to an endoplasmic reticulum (ER) stress-mediated cancer cell death. This antitumor action of arginine depletion is potentiated when combined with drugs targeting ER, such as the alkylphospholipid analog edelfosine, thus providing new insights into the role of neutrophils in cancer therapy.

## Results

### Arginine deprivation results in apoptotic cancer cell death

**Figure 1:**
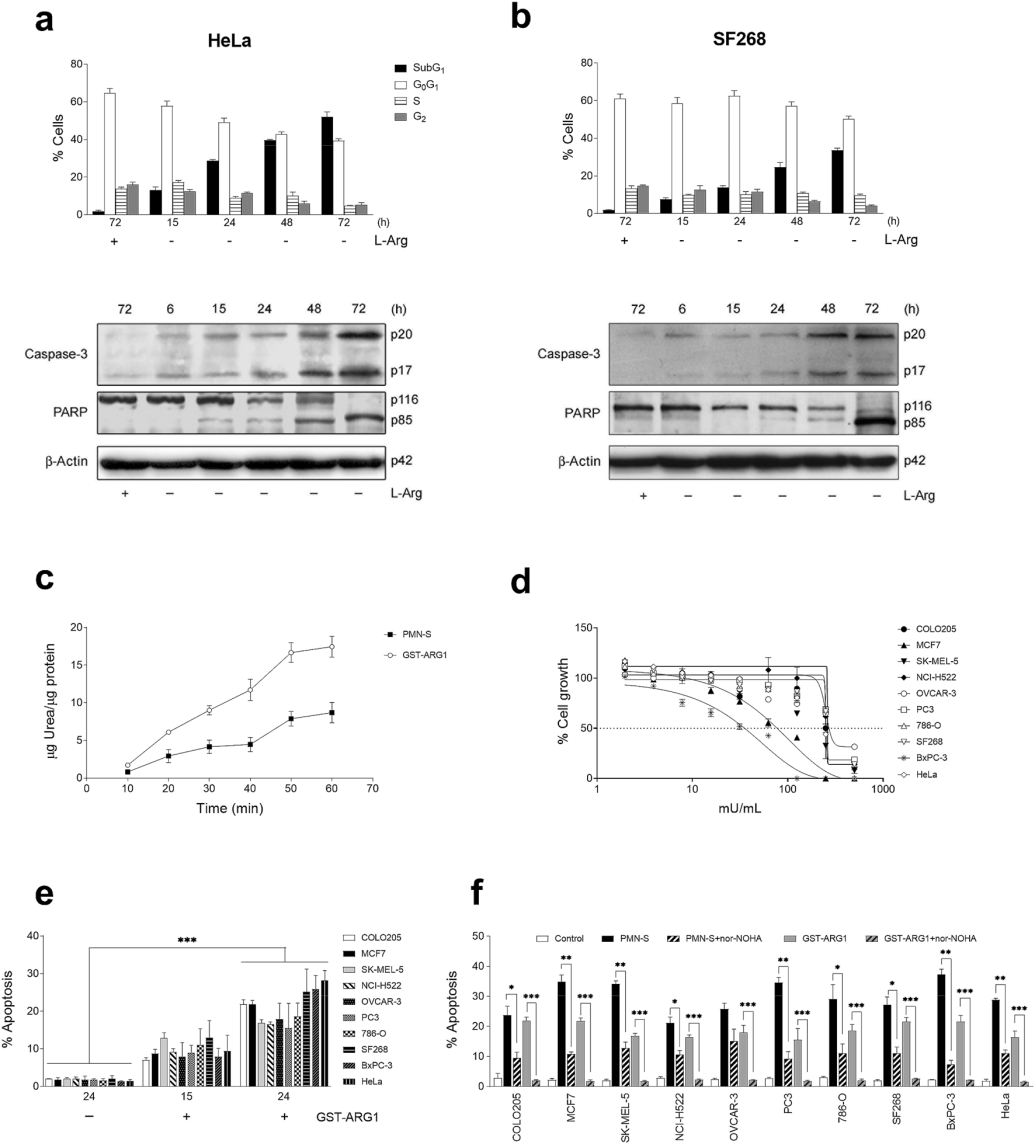
Induction of apoptosis in cancer cells grown in arginine-free culture medium and following arginine depletion by neutrophil arginase. HeLa (**a**) and SF268 (**b**) were cultured in RPMI 1640 medium with (+) and without (−) L-Arg. At the indicated times, the cell cycle was evaluated by flow cytometry. Caspase-3 activation and PARP-1 (PARP) cleavage were evaluated by Western blot. Molecular weights (in kilodaltons) of each protein are indicated at the right side of each panel. β-Actin was used as loading control. (**c**) Arginase activity of the recombinant neutrophil protein GST-ARG1 and neutrophil sonicate (PMN-S). Urea generation (μg urea produced/μg protein) was quantified every 10 min up to a total of 60 min. (**d**) A dose–response curve for the GST-ARG1 effect on cell proliferation of the indicated cell lines for 72 h was determined using the XTT assay. (**e**) Induction of apoptosis in the indicated cell lines following incubation in the absence or presence of 300 mU/ml GST-ARG1 for 15 and 24 h. (**f**) Induction of apoptosis in the indicated cell lines following incubation with 300 mU/ml of PMN-S or GST-ARG1 for 24 h in the presence or absence of the nor-NOHA arginase inhibitor (50 μM). The percentage of apoptotic cells was determined by flow cytometry (**e**,**f**). The gels were cropped to show the relevant sections. The data are representative of three independent experiments or shown as mean ± SD of three independent experiments. Asterisks indicate significant differences. *P ˂ 0.05; **P ˂ 0.01; ***P ˂ 0.001. (GraphPad Prism 8.0.1—proprietary commercial software—https://www.graphpad.com/).

The human cervical epithelial carcinoma HeLa cell line as well as the human glioblastoma SF268 cell line underwent apoptosis when grown in arginine-deficient culture medium, as assessed by an increase in the percentage of hypodiploid cells in flow cytometry analysis, due to DNA fragmentation (Fig. 1a,b). The induction of apoptosis was also assessed biochemically by caspase-3 activation, following its cleavage into the p20 and p17 fragments, as well as the cleavage of the typical caspase-3 substrate poly(ADP-ribose) polymerase-1 (PARP-1), using a polyclonal anti-human caspase-3 antibody that recognized the active caspase-3 forms, and the anti-PARP C2.10 monoclonal antibody that detected both the 116 kDa intact form and the 85 kDa cleaved form of PARP-1 (Fig. 1a,b).

### Human neutrophil arginase-1 depletes arginine and induces apoptosis in cancer cells

Human neutrophils constitutively express high amounts of arginase-1^8^. We next disrupted human neutrophils by sonication and determined arginase activity in a sonicate of PMNs (PMN-S). Using human PMNs from different blood donors, mean arginase activity of the PMN-S was 1605 ± 616 mU/mg protein (*n* = 6), in agreement with previous estimates: 1644 ± 423 mU/mg protein following 0.5% Triton X-100-mediated solubilization^8^, and 1550 ± 459 mU/mg protein after cell sonication^9^. Addition of PMN-S (300 or 500 mU/ml arginase) rapidly depleted arginine (˂ 3 µM) from culture medium after 45 min incubation, in agreement with previous estimates^9^, in which arginine content in culture medium was reported to be totally depleted after incubation with PMN-S (300 mU/ml arginase activity), and T-cell proliferation was completely prevented after only 1 h incubation with PMN-S (100 to 200 mU/ml arginase activity). An arginase activity of 300 mU/ml corresponded to the cellular content of about 3.6 ± 0.5 × 10^6^ PMNs per milliliter. PMN-S induced apoptosis in human cancer cells after 24 h incubation (28.6 ± 0.6% apoptosis in HeLa cells, and 26.7 ± 2.3% apoptosis in SF268 cells), and this apoptotic response was inhibited at a great extent (82% and 62% inhibition in HeLa and SF268 cells, respectively) by preincubation with 50 µM of the specific and potent arginase inhibitor *N*-ω-hydroxy-nor-l-arginine (nor-NOHA)^19^, which has been previously shown to efficiently block the loss of arginine induced by PMN-S^8,9^. These results suggest that neutrophil arginase-1 represents a major component of the PMN-S that is able to induce apoptosis in cancer cells.

Neutrophil differentiation is accompanied by a down-regulation of genes involved in RNA processing^20^, which may underlie, at least in part, the rather high expression of alternative splicing-derived isoforms in terminal differentiated peripheral blood human neutrophils^21–23^. Following cloning of arginase by RT-PCR from the isolated mRNA of peripheral blood human neutrophils, its sequence was identical to that of human liver arginase-1 (GenBank/European Molecular Biology Laboratory database accession no. NM_000045). Thus, neutrophils did not express a new isoform of arginase. A recombinant GST-neutrophil arginase-1 protein was generated as a ~ 66-kDa protein (Supplementary Fig. S1a). A specific polyclonal rabbit antibody to arginase-1^8^ recognized neutrophil arginase-1 from a neutrophil extract, and the recombinant GST-arginase-1 (GST-ARG1) after being expressed in *E. coli*, as bands of about 34 and 66 kDa, respectively (Supplementary Fig. S1b). Recombinant GST-ARG1 showed a higher specific activity than PMN-S (Fig. 1c), inhibited cell proliferation (Fig. 1d), and induced apoptosis (Fig. 1e) when incubated with different human cancer cell lines. Using the same enzyme activity units, the apoptotic activity exerted by GST-ARG1 was lower than that induced by PMN-S (Fig. 1f), suggesting that neutrophil sonicates could contain additional components detrimental to malignant cells. To investigate the contribution of arginase-mediated arginine depletion in this proapoptotic action, we used the specific arginase inhibitor nor-NOHA. This latter by itself did not promote apoptosis in any cell line tested (˂ 4% in all cases), but blocked arginine depletion^9^, markedly prevented the apoptotic cell death of tumor cells (over 88% inhibition) induced by recombinant human neutrophil arginase-1 (Fig. 1f), and inhibited significantly and to a great extent the apoptosis response induced by PMN-S in a number of human cancer cell lines derived from different tissues. Because nor-NOHA did not completely abrogate the above apoptotic response induced by PMN-S in cancer cells, additional neutrophil constituents are suggested to display detrimental activities to cancer cells, albeit the release of neutrophil arginase seems to constitute the major event promoting arginine depletion and subsequent cancer cell death.

### Differential sensitivity of human cancer cells to arginase activity

**Figure 2:**
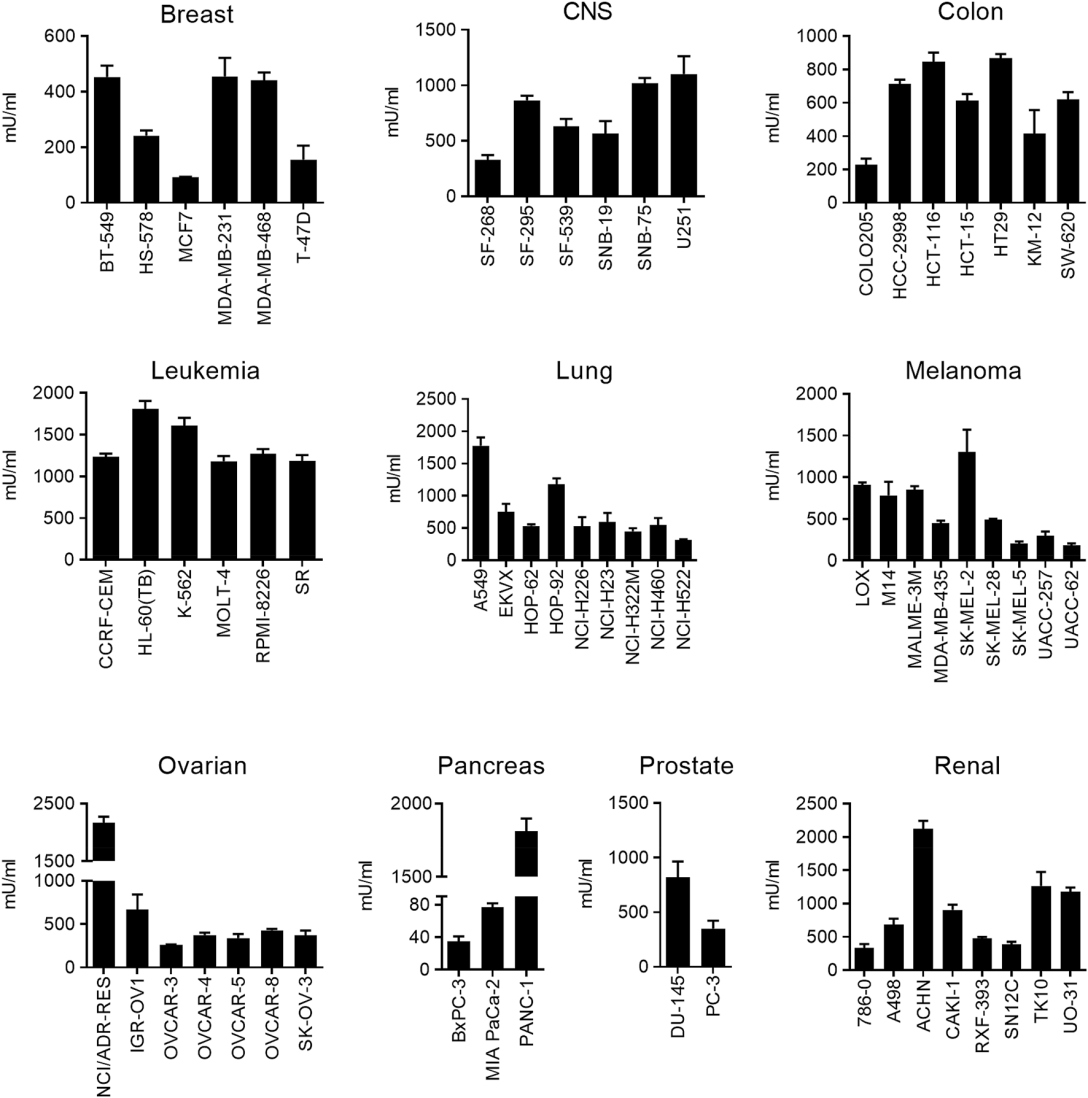
GST-ARG1 IC_50_ values (mU/ml) in human tumor cell line growth inhibition assays. Different cell lines, derived from the indicated tissues, were cultured for 72 h with different concentrations of GST-ARG1, and then cell proliferation was determined with the XTT assay. Data correspond to the means ± SD of the IC_50_ values for each cell line of at least three independent experiments performed in triplicate. CNS: Central Nervous System. (GraphPad Prism 8.0.1—proprietary commercial software—https://www.graphpad.com/).

In order to determine which cancer cell types are more sensitive to the arginase activity, leading to arginine deprivation, we determined, by the XTT method, the cell growth inhibition profile of GST-ARG1 using the National Cancer Institute (NCI)-60 cancer cell line panel as well as several human pancreatic ductal adenocarcinoma cell lines. The IC_50_ (50% inhibitory concentration) data of the GST-ARG1 in the above cell lines are shown in Fig. 2. The addition of GST-ARG1 leads to cell growth inhibition in most of the cells in a concentration-dependent manner, with pancreatic, breast, ovarian and lung cancer cells displaying, as a whole, a higher sensitivity to arginine depletion. Leukemic cells were the least sensitive cells to GST-ARG1, whereas a number of different solid-derived tumor cells showed sensitivity to less than 400 mU/ml GST-ARG1 (Fig. 2). GST-ARG1 exhibited a very potent inhibitory activity, with an IC_80_ (80% inhibitory concentration) of less than 400 mU/ml, in some human cell lines, namely: pancreatic cancer BxPC-3 (84.9 mU/ml), breast cancer MCF7 (163.5 mU/ml), cervical cancer HeLa (373.2 mU/ml), and melanoma SK-MEL-5 (378.2 mU/ml) (Supplementary Table S1). Interestingly, pancreatic cancer BxPC-3 cells were very sensitive to the action of arginase-1, either as a recombinant enzyme or from neutrophil sonicates (Figs. 1f, 2). About 82% of the apoptotic response induced by PMN-S in BxPC-3 was due to arginase activity, as estimated by the level of cell death inhibition by the arginase inhibitor nor-NOHA (Fig. 1f).

### Arginase released from activated human neutrophils kills preferentially cancer cells over normal cells

Because the addition of human neutrophil sonicates could be considered somewhat artificial as some of the antitumor molecules present in the sonicate might not be released by neutrophils normally, we decided next to investigate the ability of different stimuli to induce secretion of arginase-1, and how this secreted arginase-1 present in conditioned medium of stimulated human neutrophils could affect cancer cell viability.

**Figure 3:**
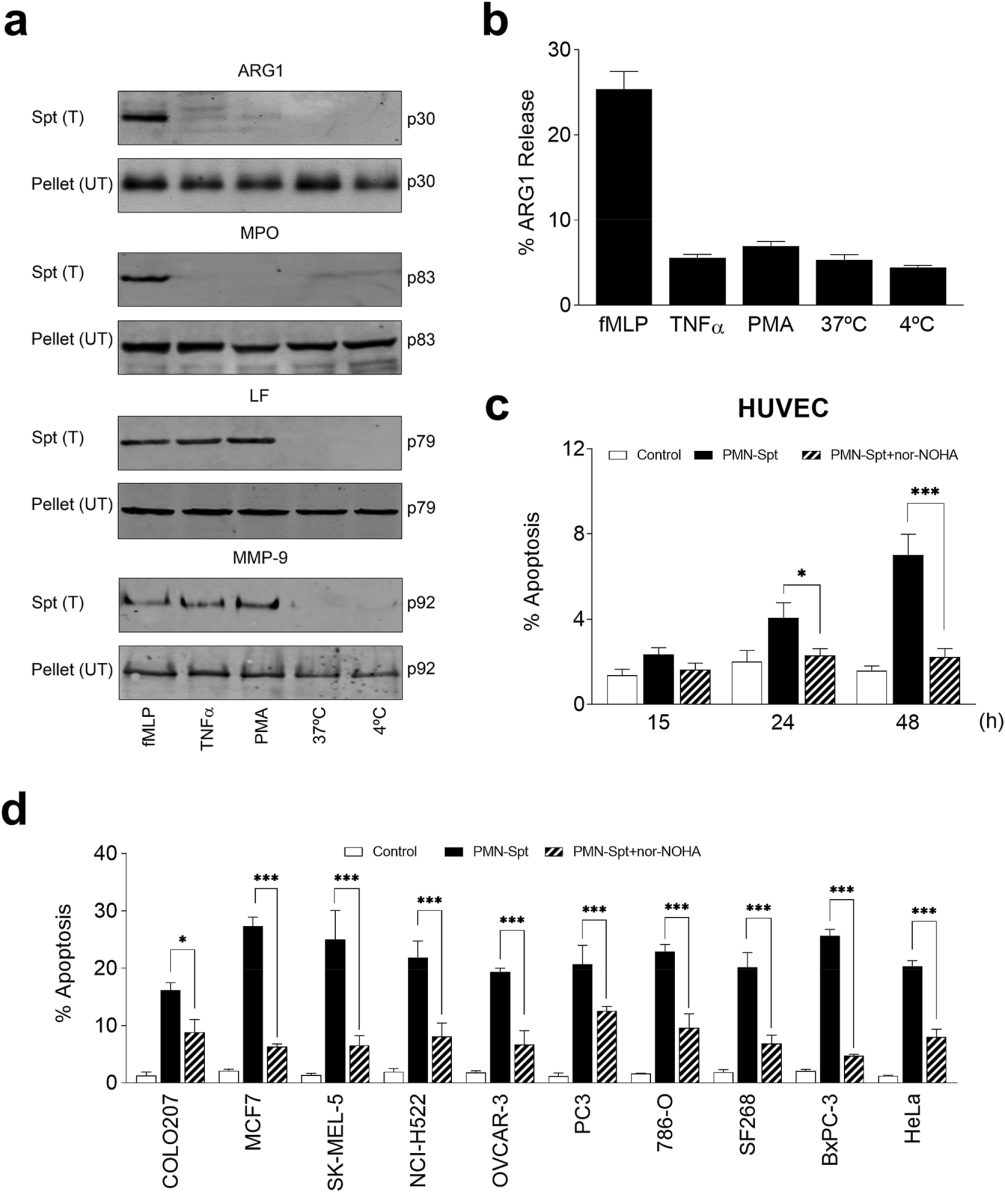
Arginase-1 is secreted in activated neutrophils and induces apoptosis in tumor cells lines. (**a**) 1.5 × 10^7^ neutrophils were incubated for 15 min at 4 °C, and at 37 °C in the absence or presence of 100 nM fMLP, 50 ng/ml TNFα or 2.5 μg/ml PMA. Cells were then pelleted by centrifugation and the secreted proteins were identified by Western blot in the supernatants of treated cells [Spt (T)]. In parallel, untreated neutrophils were also centrifuged, and pellets [Pellet (UT)] were analyzed for the indicated proteins to assure the presence of similar protein content before stimulation. Molecular weights (in kilodaltons) of each protein are indicated at the right side of the panel. *ARG1* arginase-1, *MPO* myeloperoxidase, *LF* lactoferrin, *MMP-9* metalloproteinase-9. Western blot images are representative of three independent experiments. (**b**) The percentage of arginase-1 (ARG1) released after neutrophil activation was determined by comparing the arginase activity present in the stimulated neutrophil supernatant with the sum of the arginase activity present in the cell pellet and the supernatant after each treatment. (**c**,**d**) 1.5 × 10^7^ neutrophils were stimulated with 100 nM fMLP, and the supernatant was co-cultured with HUVEC cells for indicated times (**c**) and with cancer cell lines for 24 h (**d**), in the absence or presence of 50 μM nor-NOHA inhibitor. The percentage of apoptosis by flow cytometry was quantified by Annexin-V staining (FlowJo X 10.0.7r2—proprietary commercial software—https://www.flowjo.com/). Tumor cells treated with supernatants from untreated neutrophils (Control) were run in parallel. The gels were cropped to show the relevant sections. The data in (**b**–**d**) correspond to the mean ± SD of at least three independent experiments. Asterisks indicate significant differences. *P < 0.05; ***P < 0.001. (GraphPad Prism 8.0.1—proprietary commercial software—https://www.graphpad.com/).

Neutrophils express constitutively high amounts of arginase-1, which is stored in granules^8^, and active neutrophil arginase-1 is released following simultaneous secretion of different cytoplasmic granules^24^. We found that arginase-1 was released from human neutrophils upon stimulation with the complete secretagogue *N*-formyl-methionyl-leucine-phenylalanine (fMLP) (100 nM) (Fig. 3a,b), which induced exocytosis of all neutrophil cytoplasmic granules, including tertiary, specific and azurophilic granules, as assessed by the release of their corresponding markers, namely myeloperoxidase (azurophilic granules), lactoferrin (specific granules) and MMP-9/gelatinase (tertiary granules) (Fig. 3a). Incubation of human neutrophils under experimental conditions (tumor necrosis α, TNFα; phorbol 12-myristate 13-acetate, PMA) that mobilized only specific and tertiary granules (Fig. 3a), released only little amounts of arginase protein as assessed by Western blot (Fig. 3a) and measurements of arginase activity (Fig. 3b). These results indicated that fMLP-stimulated human neutrophils released significant amounts of neutrophil arginase-1, and therefore supernatant (conditioned medium) from fMLP-stimulated human neutrophils (PMN-Spt) was used in the subsequent experiments to examine the effect of released neutrophil arginase-1 on cancer cells. PMN-Spt, derived from 1.5 × 10^7^ human fMLP-stimulated neutrophils, led to arginase-1 activities of about 315 ± 22.6 mU/ml that were found enough to deplete L-Arg levels (˂ 3 µM) in the extracellular medium.

Incubation of a primary culture of human umbilical vein endothelial cells (HUVEC) with PMN-Spt induced a weak apoptosis response in HUVEC after prolonged incubation times (4 and 7% apoptosis after 24 and 48 h incubation, respectively) that was inhibited by nor-NOHA (Fig. 3c). However, incubation with PMN-Spt for 24 h induced a potent apoptosis in a number of cancer cell lines from different tissues, and this apoptotic response was inhibited by incubation with the specific arginase inhibitor nor-NOHA (Fig. 3d). Thus, in line with the above experiments using PMN-S, these results strongly suggest that arginase-1, released from activated neutrophils, depletes arginine in the extracellular milieu, and this leads eventually to cancer cell apoptosis. Interestingly, these results indicate that human cancer cells are more sensitive than normal HUVEC to human neutrophil arginase-mediated arginine deprivation (cf. Fig. 3c,d).

### Released neutrophil arginase induces apoptosis in cancer cells through an endoplasmic reticulum (ER) stress-mediated process

**Figure 4:**
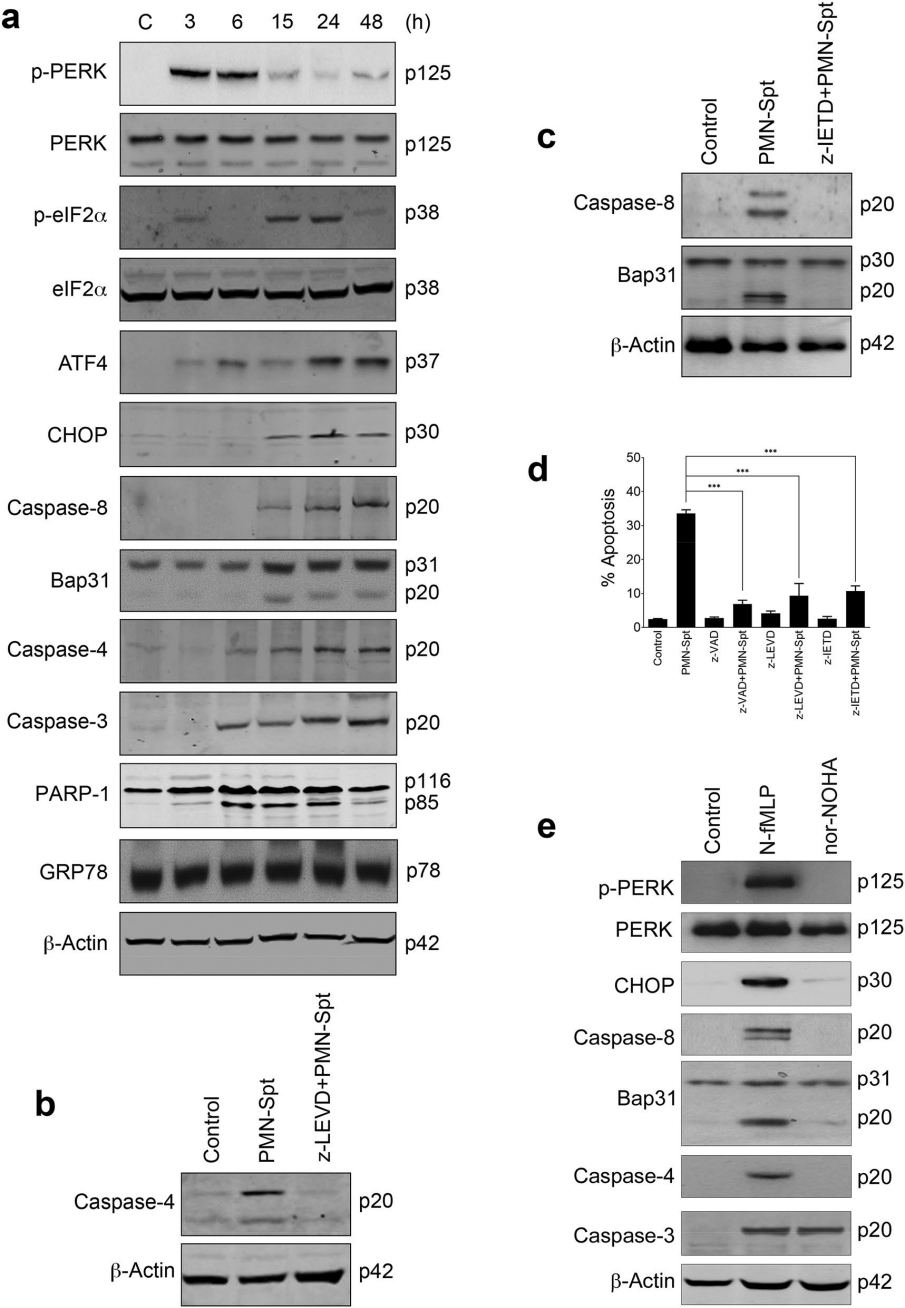
Conditioned media from activated neutrophils induce apoptosis in HeLa cancer cells by activation of ER stress signals. (**a**) 1.5 × 10^7^ neutrophils were stimulated with 100 nM fMLP, and the supernatants were incubated with HeLa cells for the indicated times, and then protein extracts were prepared and analyzed by Western blot. (**b**) Supernatant from fMLP-stimulated neutrophils (PMN-Spt) was incubated with HeLa cells for 48 h, which were previously untreated or pretreated for 1 h with 20 μM z-LEVD-fmk (caspase-4 inhibitor), and then analyzed for caspase-4 activation. (**c**) Supernatant from fMLP-stimulated neutrophils (PMN-Spt) was incubated with HeLa cells for 24 h, which were previously untreated or pretreated for 1 h with 100 μM z-IETD-fmk (caspase-8 inhibitor), and then analyzed for caspase-8 activation and Bap31 cleavage. (**d**) Induction of apoptosis determined by Annexin-V staining following incubation of HeLa cells with PMN-Spt for 24 h, in the absence or presence of z-VAD, z-LEVD or z-IETD (FlowJo X 10.0.7r2—proprietary commercial software—https://www.flowjo.com/). The data represented correspond to the mean ± SD of three independent experiments. Asterisks indicate significant differences. ***P < 0.001. (**e**) 1.5 × 10^7^ neutrophils were stimulated with 100 nM of fMLP, and the supernatants (PMN-Spt) were incubated for 24 h with HeLa cells in the absence or presence of 50 μM nor-NOHA. Protein extracts were analyzed by Western blot for the indicated proteins. β-Actin was used as loading control. Molecular weights (in kilodaltons) of each protein are indicated at the right side of the panel. The gels were cropped to show the relevant sections. The results shown in (**a**–**c**,**e**) are representative of three independent experiments. (GraphPad Prism 8.0.1—proprietary commercial software—https://www.graphpad.com/).

PMN-Spt from human neutrophils stimulated with 100 nM fMLP induced an endoplasmic reticulum (ER) stress response in HeLa cervical cancer cells as assessed by the analysis of a number of ER stress-associated markers, including phosphorylation of PERK (p-PERK), phosphorylation of eukaryotic translation initiation factor 2α-subunit (p-eIF2α) and activation of caspase-4 (Fig. 4a). This ER stress response preceded the induction of apoptosis as assessed biochemically by caspase-3 activation and caspase-3-mediated PARP-1 cleavage (Fig. 4a). Incubation of HeLa cells with PMN-Spt induced an increase in C/EBP homologous protein (CHOP) expression (Fig. 4a). CHOP, also known as DNA damage-inducible transcript 3 or GADD153, is a regulator and marker for ER stress-induced apoptosis, and CHOP upregulation has been involved in the triggering of ER stress-mediated apoptosis^25^. In addition, caspase-8 was activated and B-cell receptor associated protein 31 (Bap31) was cleaved following incubation with PMN-Spt (Fig. 4a). Bap31 is an ER membrane protein, and caspase-8-mediated cleavage of Bap31 into the p20 fragment directs proapoptotic signals between the ER and mitochondria^26^. Nevertheless, expression of GRP78/BiP, a major ER chaperone and a master regulator of the unfolded protein response (UPR) involved in cell survival^27^, was not affected by PMN-Spt (Fig. 4a). Preincubation of HeLa cells with the specific inhibitor for caspase-4 z-LEVD-fmk inhibited caspase-4 activation (Fig. 4b), and the specific inhibitor for caspase-8 z-IETD-fmk blocked activation of caspase-8 and Bap31 cleavage (Fig. 4c). Both z-LEVD-fmk and z-IETD-fmk inhibited apoptosis following incubation of HeLa cells with PMN-Spt (Fig. 4d). Preincubation with the pan-caspase inhibitor z-VAD-fmk prevented apoptosis triggered by PMN-Spt at even a higher degree (Fig. 4d), suggesting that additional caspases could participate in the above apoptotic response. In this regard, caspase-3, a major executioner caspase required for most of the typical hallmarks of apoptosis, including DNA degradation and chromatin condensation^28^, was activated after 6 h incubation with PMN-Spt (Fig. 4a). Preincubation of HeLa cells with nor-NOHA inhibited the ER stress response induced by PMN-Spt, as assessed by the inhibition of PERK phosphorylation, CHOP upregulation, caspase-8 and -4 activation, and Bap31 cleavage (Fig. 4e). This indicates that the arginase activity present in PMN-Spt was responsible for the induction of ER stress in HeLa cells. However, preincubation of HeLa cells with nor-NOHA did not prevent caspase-3 activation (Fig. 4e), suggesting that PMN-Spt contains additional molecules able to activate caspase-3 and to promote a secondary and less potent apoptotic response (Fig. 3d).

**Figure 5:**
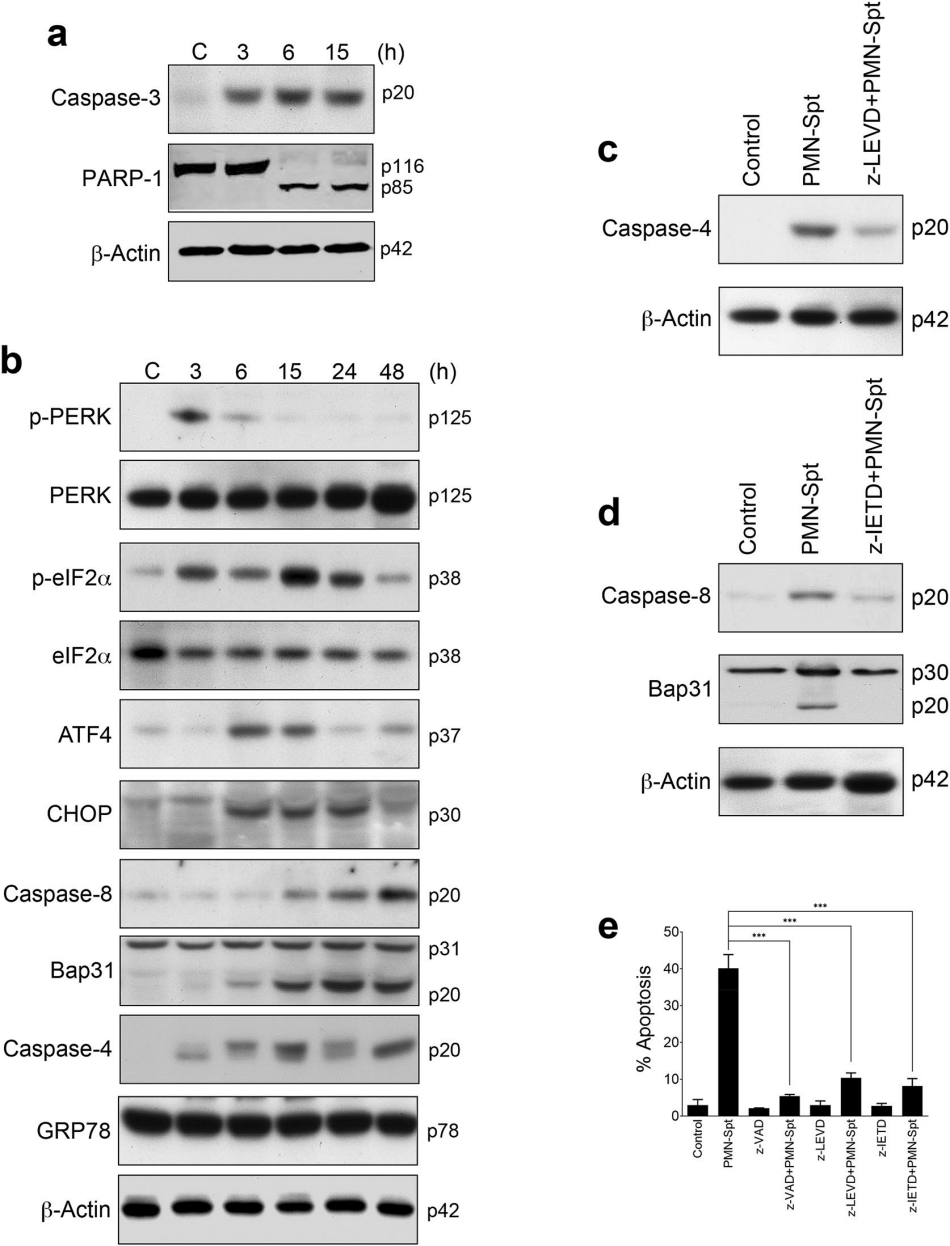
Conditioned media from activated neutrophils induce apoptosis in Bx-PC-3 pancreatic tumor cells by activation of ER stress signals. (**a**,**b**) 1.5 × 10^7^ neutrophils were stimulated with 100 nM fMLP, and the supernatant was incubated with the BxPC-3 cell line for the indicated times. Then, protein extracts were prepared and analyzed by Western blot. (**c**,**d**) Supernatants from fMLP-stimulated neutrophils (PMN-Spt) were incubated with BxPC-3 pancreatic cancer cells for 24 h, which were previously untreated or pretreated for 1 h with 20 μM z-LEVD-fmk (caspase-4 inhibitor) or 100 μM z-IETD-fmk (caspase-8 inhibitor), and then analyzed for caspase-4 activation (**c**), or caspase-8 activation and Bap31 cleavage (**d**). β-Actin was used as loading control. Molecular weights (in kilodaltons) of each protein are indicated at the right side of each panel. The results shown in (**a**–**d**) are representative of three independent experiments. The gels were cropped to show the relevant sections. (**e**) Induction of apoptosis determined by Annexin-V staining following incubation of BxPC-3 cells with PMN-Spt for 24 h, in the absence or presence of z-VAD, z-LEVD or z-IETD (FlowJo X 10.0.7r2—proprietary commercial software—https://www.flowjo.com/). The data correspond to the mean ± SD of at least three independent experiments. Asterisks indicate significant differences. ***P < 0.001. (GraphPad Prism 8.0.1—proprietary commercial software—https://www.graphpad.com/).

Pancreatic cancer cells behaved as the most sensitive cancer cells to arginase incubation (Fig. 2 and Supplementary Table S1). PMN-Spt induced a potent apoptosis in human BxPC-3 pancreatic cancer cells (Fig. 3d), which was accompanied by a rapid caspase-3 activation and subsequent PARP-1 cleavage, a well-known caspase-3 substrate and a biochemical marker of apoptosis^29^, after only 3–6 h incubation (Fig. 5a). Thus, we next analyzed the putative involvement of ER stress in the apoptotic response induced by PMN-Spt in BxPC-3 pancreatic cancer cells. A rapid increase in the phosphorylation of PERK and eIF2α, as well as a remarkable upregulation of ATF4 and CHOP, suggested that PMN-Spt incubation led to a potent ER stress response in these pancreatic cancer cells (Fig. 5b). Caspase-4, functioning as an ER stress-specific caspase involved in apoptosis in humans^30^, was rapidly activated by PMN-Spt, and the specific caspase-4 inhibitor z-LEVD-fmk inhibited caspase-4 processing (Fig. 5b,c). Likewise, PMN-Spt induced processing of caspase-8 that was inhibited by the specific caspase-8 inhibitor z-IETD-fmk (Fig. 5b,d). This caspase-8 inhibition blocked PMN-Spt-induced Bap31 cleavage (Fig. 5b,d). The use of the specific inhibitors z-LEVD-fmk (caspase-4 inhibitor) and z-IETD-fmk (capase-8 inhibitor) strongly inhibited the apoptotic response induced by PMN-Spt as determined by Annexin-V staining by flow cytometry (Fig. 5e). The pan-caspase inhibitor z-VAD-fmk was more efficient than the above specific inhibitors for caspase-4 and -8, in almost totally preventing the apoptotic response following PMN-Spt incubation (Fig. 5e).

### PERK signaling in ER stress-mediated pancreatic cancer cell death by neutrophil arginase

**Figure 6:**
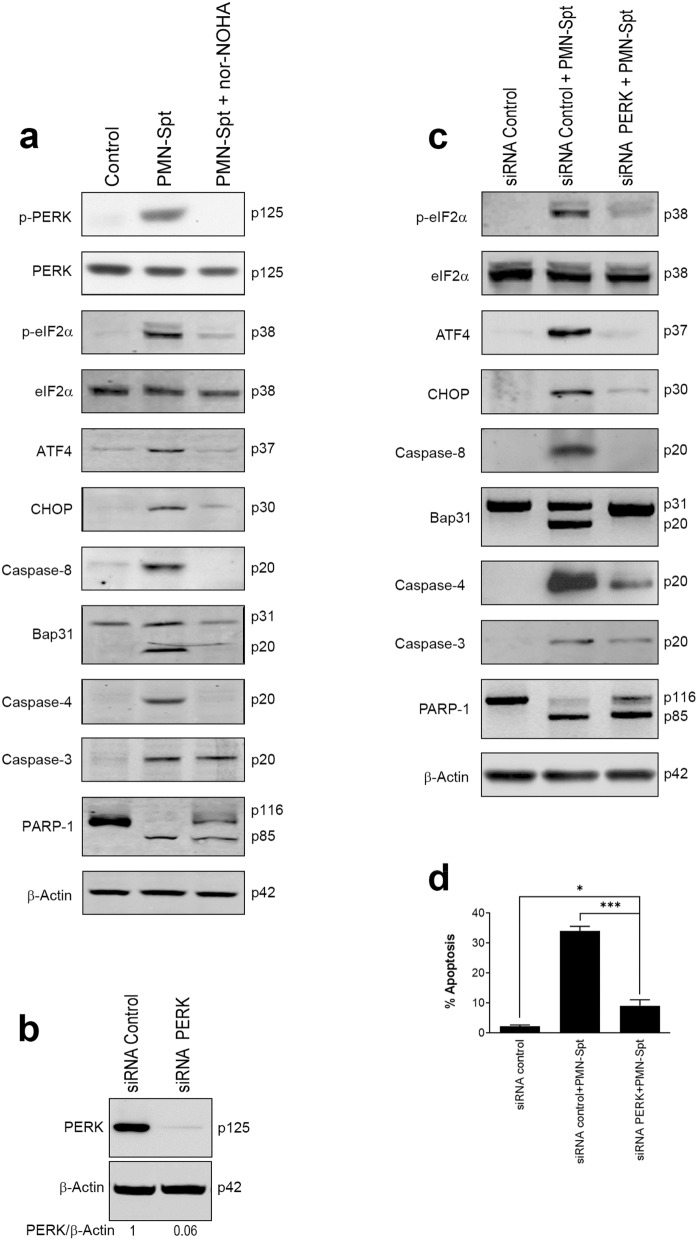
Arginase released from activated neutrophils induces an ER stress response in BxPC-3 pancreatic cancer cells leading to cell death. (**a**) 1.5 × 10^7^ neutrophils were stimulated with 100 nM of fMLP, and the supernatant (PMN-Spt) was incubated with BxPC-3 cells for 24 h in the absence or presence of 50 μM nor-NOHA. Protein extracts were prepared and analyzed by Western blot for the indicated proteins. (**b**–**d**) PERK silencing inhibits PMN-Spt-induced ER stress and apoptosis in BxPC-3 cells. BxPC-3 cells were transiently transfected with 50 nM control siRNA and PERK siRNA, and silencing was verified by Western blot (**b**). BxPC-3 cells transfected with PERK siRNA were incubated with PMN-Spt for 24 h and analyzed by Western blot for the indicated proteins involved in ER stress and apoptosis. β-Actin was used as loading control. The gray value of PERK band was normalized against its internal control β-actin, and expressed as values relative to the control to measure relative PERK abundance (**b**). Molecular weights (in kilodaltons) of each protein are indicated at the right side of each panel. The results shown in (**a**–**c**) are representative of three independent experiments. The gels were cropped to show the relevant sections. (**d**) Following 24 h transfection with PERK siRNA, cells were incubated with PMN-Spt for 24 h and the percentage of apoptotic cells was quantified by flow cytometry (FlowJo X 10.0.7r2—proprietary commercial software—https://www.flowjo.com/). The data shown correspond to the mean ± SD of three experiments. Asterisks indicate significant differences. *P < 0.05; ***P < 0.001. (GraphPad Prism 8.0.1—proprietary commercial software—https://www.graphpad.com/).

The above results suggest that ER stress plays a major role in the induction of a caspase-mediated apoptosis in cancer cells by PMN-Spt. The first indication for the onset of ER stress following the action of PMN-Spt was detected after only 3 h incubation, as assessed by PERK phosphorylation, before subsequent caspase activation (Figs. 4a, 5a). Preincubation with nor-NOHA blocked the above ER stress response in PMN-Spt-treated BxPC-3 pancreatic cancer cells, inhibiting drastically PERK and eIF2α phosphorylation, as well as ATF4 and CHOP upregulation (Fig. 6a). Caspase-8 and -4 were inhibited by preincubation with nor-NOHA (Fig. 6a). These results strongly suggest that arginase-1 activity, secreted in PMN-Spt, is critically involved in the ER stress response triggered by PMN-Spt in BxPC-3 pancreatic cancer cells. Despite nor-NOHA inhibited drastically the apoptotic and ER stress responses in BxPC-3 cells following treatment with PMN-Spt, caspase-3 activation and PARP-1 cleavage could still be observed (Fig. 6a). PARP-1 cleavage was partially inhibited but not blocked, suggesting that this caspase-3 activation could account for the remaining apoptotic response triggered by PMN-Spt, independently of arginase-1 release, in the presence of nor-NOHA (Fig. 3d).

We next analyzed, through RNA silencing, the role of PERK in the ER stress and apoptotic response triggered by PMN-Spt. BxPC-3 cells transfected with *PERK* siRNA downregulated PERK expression (Fig. 6b), and *PERK* silencing drastically inhibited all the typical ER stress markers (peIF2α phosphorylation, ATF4 and CHOP upregulation, caspase-8 activation and Bap31 cleavage) (Fig. 6c), and the apoptotic response induced by PMN-Spt (Fig. 6d). Caspase-4 was strongly inhibited, whereas caspase-3 activation and PARP-1 cleavage were diminished by *PERK* silencing in PMN-Spt-treated cells, but not totally prevented (Fig. 6c). The inhibitory action of *PERK* silencing on the different ER stress markers indicates that that PERK is required for ER stress following arginase-1-mediated depletion of L-Arg in the culture medium. Taken together, these results strongly indicate PMN-Spt induces apoptosis in pancreatic cancer cells mainly through an arginase-1-dependent L-Arg depletion and subsequent PERK-driven ER stress. The small percentage of apoptosis observed after PERK silencing in PMN-Spt-treated BxPC-3 cells (Fig. 6d) suggests that PMN-Spt also contains some additional molecules that could promote a low apoptotic response via caspase-3 activation (Fig. 6c,d).

### Arginine deprivation potentiates apoptosis induced by the ER-targeted alkylphospholipid edelfosine

**Figure 7:**
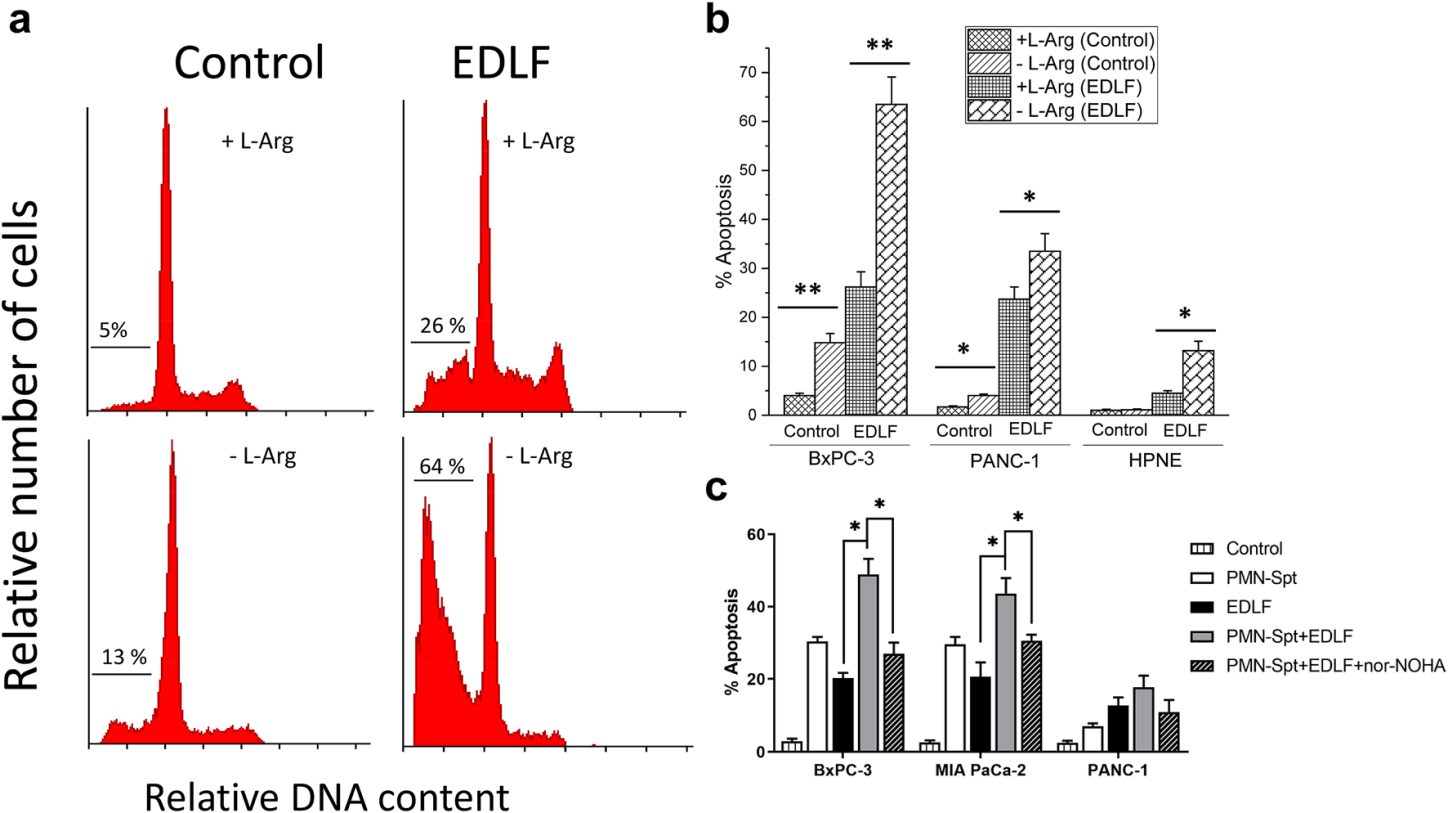
Arg deprivation potentiates the apoptotic action of ER-targeted edelfosine in human pancreatic cancer cells. (**a**) BxPC-3 cells, in L-Arg-containing (+ L-Arg) or L-Arg-deficient (− L-Arg) culture medium, were incubated in the absence (Control) or presence of 20 µM edelfosine (EDLF) for 48 h, and then analyzed for cell cycle profiling. Representative histograms, from at least three different experiments, are shown, and the percentages of cells at sub-G_0_/G_1_ (apoptosis) are indicated in each cell cycle profile. (**b**) Different human pancreatic cancer cell lines (BxPC-3, PANC-1) and non-tumorigenic human pancreatic HPNE cell line, in L-Arg-containing (+ L-Arg) or L-Arg-deficient (− L-Arg) culture medium, were incubated in the absence (Control) or presence of 20 µM edelfosine (EDLF) for 48 h, and then apoptosis was analyzed by flow cytometry as the percentage of hypodiploid cells (sub-G_0_/G_1_) following cell cycle analysis. (**c**) 1.5 × 10^7^ neutrophils were stimulated with 100 nM fMLP, and the supernatant was co-cultured with the human pancreatic cell lines BxPC-3, MIA PaCa-2 and PANC-1 in L-Arg containing culture medium for 48 h in the absence (PMN-Spt) or presence of 20 µM edelfosine (PMN-Spt + EDLF) or 50 µM nor-NOHA plus 20 µM edelfosine (PMN-Spt + EDLF + nor-NOHA). Untreated cells (Control) and cells treated only with 20 µM edelfosine (EDLF) were run in parallel. After treatment, the percentage of apoptosis was determined by Annexin-V staining by flow cytometry (FlowJo X 10.0.7r2—proprietary commercial software—https://www.flowjo.com/). Data shown in (**b**,**c**) are means ± S.D. of three independent determinations. *P < 0.05; **P < 0.01. (GraphPad Prism 8.0.1—proprietary commercial software—https://www.graphpad.com/).

The alkylphosphocholine analog edelfosine accumulates in the ER and promotes apoptosis in several solid tumor cells, including pancreatic cancer cells^31^, through an ER stress pathway^31–33^. Thus, we next examined whether L-Arg depletion could potentiate the proapoptotic activity of the ER-targeted edelfosine in pancreatic cancer cells. Figure 7a shows that the ability of edelfosine to induce apoptosis in human BxPC-3 pancreatic cancer cells was highly potentiated when cultured in L-Arg-deficient medium. Furthermore, this potentiating effect of L-Arg depletion on edelfosine-induced apoptosis was also observed in human PANC-1 pancreatic cancer cells (Fig. 7b), which show a higher resistance to several chemotherapeutic agents (gemcitabine, 5-fluorouracil, and cisplatin) used in pancreatic cancer patients^34^. In contrast, incubation of the non-tumorigenic pancreatic cell line hTERT-HPNE (hTERT-immortalized human pancreatic nesting expressing cell line)^35^ in a L-Arg-deficient culture medium slightly potentiated the weak proapoptotic effect of edelfosine, and the rate of apoptosis was significantly lower when compared to that of tumorigenic pancreatic cancer cell lines (Fig. 7b). Furthermore, the combination of PMN-Spt and edelfosine also led to a clear increase in the percentage of apoptosis in different human pancreatic cancer cells, and this potentiating effect was prevented by preincubation with nor-NOHA (Fig. 7c).

## Discussion

The results reported here provide the first demonstration that neutrophil exocytosis can induce ER stress-mediated apoptosis in cancer cells via the enzymatic activity of exocytosed arginase-1, which rapidly depletes arginine in the surrounding medium. Human neutrophils act as a double-edge sword in some pathologies, such as cancer. Neutrophils have been implicated as playing a role of immune surveillance against cancer and as a facilitating factor in cancer progression^3,36,37^. A high level of neutrophil-to-lymphocyte ratio in the blood of cancer patients and neutrophil infiltration within tumors have been associated with poor clinical outcome^6,38^. Neutrophils have long been found in different types of tumors, and these tumor-associated neutrophils (TANs) can show antitumor (TAN1) or pro-tumor (TAN2) activity^39^, reminiscent of the M1/M2 polarization of macrophages. These apparently different neutrophil phenotypes have been recently proposed to derive from the same neutrophil population, as a result of a differential granule mobilization following different stages of priming and activation^3^.

Neutrophils recruited at the tumor site contain a complete weaponry to destroy tumor cells, including proteases, membrane-perforating agents, soluble cell killing mediators, generation of reactive oxygen species and hypochlorous acid, and contribute to antibody-mediated tumor cells destruction through the expression of several Fc receptors^40^. In this regard, neutrophils are able to kill antibody opsonized cancer cells through a new way of cell death named as trogoptosis^41^, which involves CD11b/CD18-dependent neutrophil-tumor cell conjugate formation, followed by an antibody-mediated trogocytosis through neutrophils exerting an active mechanical disruption of the cancer cell plasma membrane, leading to a lytic (i.e., necrotic) type of cancer cell death. CD11b/CD18 is mainly located in tertiary granules in resting human neutrophils and it is upregulated at the cell membrane of activated neutrophils upon granule secretion^42^. Here, we report a new way by which neutrophils could kill tumor cells, namely by releasing arginase-1, either by exocytosis or following cell demise. Thus, granule secretion seems to be critical in the killing activity of neutrophils on tumor cells. Neutrophil exocytosis is tightly regulated through the interaction of SNARE proteins^2^, by mechanisms that are not yet fully elucidated.

**Figure 8:**
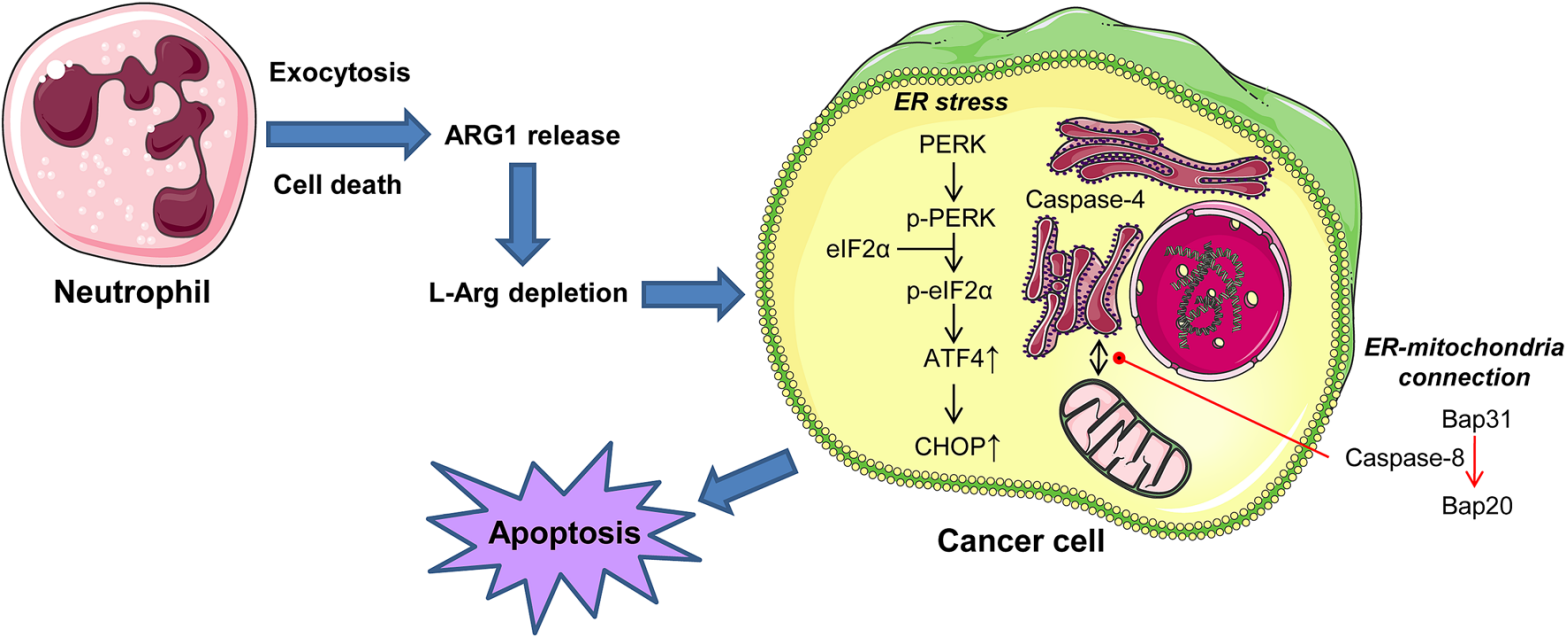
Schematic model of ER involvement in the induction of apoptosis in cancer cells following release of arginase-1 from human neutrophils. This is a schematic diagram to portray a plausible mechanism of how neutrophils can induce apoptosis in tumor cells following arginase-1 (ARG1) release. ARG1 is released from human neutrophils, either after cell activation leading to exocytosis of granule contents or after cell death. ARG1 leads to L-Arg depletion in the surrounding medium, and this induces an ER stress response and apoptosis in cancer cells. During this process, caspase-4 and -8 are activated, and there is an ER-mitochondria interplay mediated by the action of caspase-8 on Bap31. See text for details. Source: Own elaboration.

So far, neutrophil arginase had been associated to its ability to suppress T cell functions^9^, thus leading to the generation of a transient immune-privileged site for the tumor^3^. However, the results reported here indicate that arginine deprivation mediated by secreted neutrophil arginase leads to ER stress and subsequent apoptosis in a wide variety of tumor cells. Figure 8 summarizes the results reported here and depicts a schematic model for the involvement of ER stress in the induction of apoptosis in cancer cells by l-arginine depletion as a result of neutrophil arginase release. Our results indicate that neutrophil arginase induces ER stress in cancer cells through PERK signaling, which involves activation of the PERK → eiF2α → ATF4 → CHOP axis that eventually leads to cell death. The PERK-ATF4-CHOP route has been shown to play a crucial role in cell death^43^. Silencing of PERK by small interfering RNA largely inhibited the ER stress and apoptosis response triggered by arginase-dependent arginine depletion. Furthermore, we found that arginine depletion by the action of neutrophil arginase led to additional markers of ER stress-mediated apoptosis, including the activation of caspase-4 and caspase-8, as well as to Bap31 cleavage, leading to the generation of the ER-localized proapoptotic Bap31-derived p20 fragment, which mediates mitochondrion-ER cross-talk through a Ca^2+^-dependent mechanism^26^.

Human neutrophils contain constitutively high levels of arginase-1^8^. Neutrophil arginase has been reported to be stored in both tertiary and azurophilic granules^8,44^, but requires the release of azurophilic granules to become fully active^24^. The presence of still unknown factors in azurophilic granules seems to be essential to provide an active neutrophil arginase at physiological pH^24^.

A number of different neutrophil-like cell populations have been reported in recent years^45^, but the cell surface markers and functions of these cell populations totally overlap with the corresponding features of circulating neutrophils, making an accurate discrimination between the above cell populations and circulating neutrophils impossible^3,46^. In this regard, myeloid-derived suppressor cells (MDSCs) have been reported to be enriched in arginase, but the seminal article^8^ that initially identified the constitutive expression of arginase-1 in human peripheral blood neutrophils showed that human circulating neutrophils expressed constitutively high amounts of arginase-1, whereas peripheral blood mononuclear cells lacked this enzyme^8^. Taking together, and according to the recently proposed novel hypothesis of neutrophil plasticity mediated by granule mobilization^3^, it could be envisaged that the above cell populations could really correspond to the same cell entity (human neutrophils), which undergo various changes in its cell surface markers and functions, depending on the local conditions, diapedesis processes, localization in blood or tissues, and cell contacts^3^. Human neutrophils highly depend on their unique and characteristic intracellular granules, which are differentially mobilized by a not yet clearly understood mechanism^2,47–49^, leading to a change in the cell surface protein profile, that could be misinterpreted as markers for novel cell entities^3^. In this context, tertiary granules, originally identified in the mid-eighties of last century^50,51^, are readily mobilized upon slight neutrophil priming^52^ and activation^53^, leading to changes in the neutrophil cell surface protein pattern and neutrophil phenotype^3,42,54^.

Because arginase-1 has been reported to be localized in cytoplasmic granules in human neutrophils^8,44^, it could be envisaged that the intracellular content of arginase-1 could vary between primed, activated or resting neutrophils. In this regard, the experimental procedure followed to isolate neutrophils from peripheral blood could affect the activation stage of these purified cells. Thus, the dextran incubation of peripheral blood, widely used as a first step in neutrophil isolation, could activate neutrophils, as assessed by an increase in cell surface CD11b^55^. CD11b/CD18 is present in the tertiary granule of human neutrophils and is readily mobilized to the cell surface upon neutrophil priming and activation^3,42,54^. The neutrophil isolation method used in this study, namely, dextran sedimentation followed by Ficoll-Hypaque gradient centrifugation of human peripheral blood neutrophil, allowed the recovery of rather high amounts of arginase-1 in isolated neutrophils as previously reported^8^. Thus, these results might suggest that part of the original arginase-1 content has been released during the cell isolation process, or that arginase-1 could be located, at least in a significant portion, in non-readily mobilized intracellular granules as previously reported, or in several localizations differentially mobilized.

There is an apparent high heterogeneity in the level of neutrophil infiltration in different tumors, some tumors being heavily infiltrated, whereas others have only moderate or low neutrophil infiltration^56^. Likewise, neutrophils have been reported to show different phenotypes with both pro-tumor and tumor-killing capacity, but it is not clear the relative abundance of these cell populations in cancer patients and illnesss severity^3,56^, albeit high levels of neutrophil-to-lymphocyte (NLR) values have been generally associated as an independent prognostic factor of poor overall survival in cancer patients^3,6^. The prognostic implications of neutrophil infiltration and relative importance of distinct phenotypes in cancer remains an open question and requires further investigation.

Because our study has been performed in vitro, the proposed model described here for a novel antitumor mechanism of neutrophils to induce cancer cell killing can be considered as a working hypothesis that should be tested and validated in the future in in vivo and clinical settings.

Although a controversial issue, some studies have reported a predisposition of neutrophils to target cancer cells, which is often leveraged to develop novel cell-based drug delivery systems^57,58^. In this regard, one of the main advantages of neutrophils is that they are present in high amounts in blood under normal circumstances, and they could theoretically outnumber and adversely affect tumor cells.

Our results indicate that pancreatic cancer cells seem to be especially sensitive to the action of arginase-mediated arginine-deprivation, and the cell demise process is mediated by a potent ER stress response. Furthermore, arginine deprivation potentiates the antitumor activity of the alkylphospholipid analog edelfosine that accumulates in the ER of pancreatic cancer cells, leading eventually to their cell demise in vitro and in vivo^31^. Taken together, our data highlight ER as a major target in cancer therapy, and could be of particularly importance in pancreatic cancer. Pancreatic adenocarcinoma responds poorly to current therapies and remains as an incurable malignancy. Pancreatic ductal adenocarcinoma is the most lethal of all common cancers, with the highest mortality-to-incidence ratio^59^. Because pancreatic cancer cells have a prominent ER^60,61^, the results reported here open a novel approach in the treatment of this incurable cancer, highlighting ER stress as a vulnerable process to be targeted in cancer therapy.

Our results suggest that in addition to being the most abundant leukocyte in blood and the body’s main guardians against infection and foreign invaders, human neutrophils could behave as a promising and appealing weapon against tumor cells through the release specific enzymes stored in their intracellular granules. The ability of directing large amounts of neutrophils to the tumor site, and the differential release of their intracellular contents in a highly regulated way, could be the underlying basis of a novel approach to treat tumors. Thus, our results support the notion that neutrophils are able not only of migrating to and infiltrating cancerous tissues promoting tumor progression^3^, but also of inducing antitumor activity by direct or indirect ways. Furthermore, the results reported here suggest that neutrophils could be a novel player to be taken into account in combination therapy. Novel insights in pharmacological regulation of the neutrophil action on tumor cells, potentiating the antitumor activity of neutrophils over their pro-tumor actions, could contribute to set up a new immunotherapeutic framework in cancer treatment, taking advantage of the most abundant leukocyte to fight cancer.

## Materials and methods

### Reagents

If not otherwise stated, chemicals were purchased from Sigma-Aldrich (St Louis, MO). Nor-NOHA was from Cayman Chemical (Ann Arbor, MI). Polyclonal rabbit anti-rat arginase-1 antiserum^8^, which is cross-reactive to mouse and human arginase-1, was kindly provided by Dr. M. Modolell (Max-Planck Institut for Immunobiology, Freiburg, Germany). Edelfosine was obtained from R. Berchtold (Biochemisches Labor, Bern, Switzerland) and stock solutions were prepared as previously described^62^. Caspase-4 inhibitor z-LEVD-fmk, and the broad pan-caspase inhibitor z-VAD-fmk were from Alexis Biochemicals (San Diego, CA). Caspase-8 inhibitor z-IETD-fmk was from Calbiochem (San Diego, CA). Acrylamide, bisacrylamide, ammonium persulfate, and *N*,*N*,*N*′*N*′-tetramethylethylenediamine were from Bio-Rad (Hercules, CA). RPMI 1640 culture medium without arginine was purchased from GIBCO-BRL (Gaithersburg, MD), and supplemented with MnCl_2_ to a physiological concentration (4 µM).

### Cell culture and arginine determination

All cell lines were from the American Type Culture Collection (ATCC, Manassas, VA), the European Collection of Authenticated Cell Cultures (ECACC, Salisbury, UK), or the Deutsche Sammlung von Mikroorganismen und Zellkulturen GmbH-DSMZ (German Collection of Microorganisms and Cell Cultures, Braunschweig, Germany). Cells were grown in RPMI 1640 or DMEM (GIBCO-BRL) containing 10% heat-inactivated fetal bovine serum (GIBCO-BRL), 2 mM l-glutamine (GIBCO-BRL), 100 U/ml penicillin, and 100 μg/ml streptomycin at 37 °C in a humidified atmosphere containing 5% CO_2_. Arginine-free culture medium was prepared by using arginine-free RPMI 1640 medium (GIBCO-BRL) and 10% dialyzed (< 10 kDa) fetal bovine serum (Sigma). Human umbilical vein endothelial cells (HUVEC) were isolated as previously described^63^. Cells were periodically tested for Mycoplasma infection and found to be negative. Arginine determination was carried out using an Agilent 1100 HPLC in conjunction with an Agilent Trap XCT mass spectrometer. ^13^C-labeled arginine was used as internal standard.

### Isolation of human neutrophils and neutrophil activation

The study was approved by the ethics committee of the Centro de Investigación del Cáncer of Salamanca, and was performed in compliance with the Declaration of Helsinki ethical principles for medical research involving human subjects. Informed consent was obtained from all participants in the study. Neutrophils were obtained from fresh human peripheral blood by dextran sedimentation and centrifugation on Ficoll-Hypaque (Pharmacia LKB Biotechnology, Uppsala, Sweden), followed by hypotonic lysis of residual erythrocytes as previously described^22^. Neutrophil activation was carried out as previously described^64^, with some modifications. For granule content release experiments 1.5 × 10^7^ freshly isolated neutrophils were incubated for 15 min at 4 °C, and at 37 °C in the absence or presence of 100 nM fMLP, 50 ng/ml TNFα or 2.5 µg/ml PMA, and then cells were pelleted by centrifugation, and the supernatants were saved for subsequent experiments and assayed for protein identification by Western blot and for arginase activity.

### Generation of neutrophil sonicates

Purified peripheral blood human PMNs were resuspended in PBS (40 × 10^6^ cells/ml), sonicated for 3 min (amplitude 80) in a Sonicator Ultrasonic Processor XL (Misonix, Inc. New Highway, Farmingdale, NY), and centrifuged at 20,000*g* for 30 min at 4 °C, as previously described^9^. Then, the supernatant was filtered (0.2 µm), protein concentration and arginase activity were determined, and aliquots were frozen at − 80 °C until use as previously described^9^.

### Arginase enzymatic assay

Arginase activity was measured as previously described^8^. To 50 µl of sample, 10 µl of 10 mM MnCl_2_ was added, and the enzyme was activated by heating for 10 min at 56 °C. Arginine hydrolysis was conducted by incubating the sample with 50 µl of 0.5 M l-arginine (pH 9.7) at 37 °C for 15–120 min. The reaction was stopped with 900 µl of H_2_SO_4_ (96%)/H_3_PO_4_ (85%)/H_2_O (1/3/7, v/v/v). The urea concentration was measured at 540 nm after addition of 20 µl of 6% α-isonitrosopropiophenone (dissolved in 100% ethanol) followed by heating at 95 °C for 30 min. One unit of enzyme activity is defined as the amount of enzyme that catalyzes the formation of 1 µmol of urea/min.

### Cell growth inhibition assay

Inhibition of cell proliferation (cytostatic activity) was determined using the XTT (sodium 3′-[1-(phenylaminocarbonyl)-3,4-tetrazolium]-bis(4-methoxy-6-nitro)-benzenesulfonic acid hydrate) cell proliferation kit (Roche Molecular Biochemicals, Mannheim, Germany) according to the manufacturer’s instructions, and as previously described with some slight modifications^65^. Cells from different tissue origin (ranging from 2500 to 6000 in 100 µl) were incubated in culture medium in the absence and in the presence of different concentrations of GST-ARG1 in 96-well flat-bottomed microtiter plates, and following 72 h of incubation at 37 °C in a humidified atmosphere of air/CO_2_ (19/1), the XTT assay was performed. Measurements were performed in triplicate, and each experiment was repeated three times. The IC_50_ and IC_80_ values (50% and 80% inhibitory concentrations), defined as the GST-ARG1 concentration required to cause 50% and 80% inhibition in cellular proliferation with respect to the untreated controls, was determined in each cell line.

### Apoptosis assay

Quantification of apoptotic cells was determined by flow cytometry as the percentage of cells in the sub-G_1_ region (hypodiploidy) in cell cycle analysis as previously described^31^. Briefly, cells (5 × 10^5^) were centrifuged and fixed overnight in 70% ethanol (MERCK, Darmstadt, Germany) at 4 °C. Then, cells were washed three times with PBS, incubated for 1 h with 1 mg/ml RNase A and 20 μg/ml propidium iodide at room temperature, and analyzed for the distinct cell cycle phases with a Becton Dickinson FACSCalibur flow cytometer. Apoptosis was also assessed using the Annexin-V/7-ADD kit (BD Biosciencies), and the whole cell population was labeled with fluorescein isothiocyanate (FITC)-conjugated Annexin-V/7-ADD without prior fixation, according to the manufacturer’s instructions. Cells were analyzed using a FACSCalibur flow cytometer (Becton Dickinson) with CellQuest Pro 4.0 software (proprietary commercial software, https://www.bd.com/en-uk/products/molecular-diagnostics/cytometric-analysis-products). At least 10,000 events were analyzed for each sample. Data analysis was carried out with FlowJo X 10.0.7r2 (Tree Star Inc., San Carlos, CA; proprietary commercial software, https://www.flowjo.com/software).

### Reverse transcriptase-polymerase chain reaction (RT-PCR)

Total RNA was extracted from human neutrophils with TRIzol Reagent (Invitrogen, Carlsbad, CA) following the manufacturer’s instructions as previously described^66^. Total RNA (5 µg), primed with oligo-dT, was reverse-transcribed into cDNA with SuperScript III First-Strand Synthesis System (Invitrogen) for RT-PCR as previously described^67^. The generated cDNA was amplified by using primers for human liver arginase-1 (NM_000045): forward- 5′-TAGAATTCATGAGCGCCAAGTCCAGA-3′; reverse-5′-TTCTCGAGCTTAGGTGGGTTAAGGTA-3′. The PCR mixture (50 µl) contained the cDNA template (1–2 µl), 10 pmol of the corresponding primers, 0.2 mM dNTP, 2.5 mM MgCl_2_, 5 units of EcoTaq DNA polymerase derived from *Thermus aquaticus* (ECOGEN, Barcelona, Spain). PCR reactions were performed in GeneAmp PCR System model 9600 (PerkinElmer, Norwalk, CT). The PCR profile was as follows: 1 cycle at 95 °C for 5 min as an initial denaturation step, then denaturation at 95 °C for 30 s, annealing at 58.5 °C for 30 s, and extension at 72 °C for 90 s (30 cycles), followed by further incubation for 15 min at 72 °C (1 cycle). An aliquot of the PCR reaction was analyzed on a 1% agarose gel in 1 × TAE (40 mM Tris–acetate, 1 mM EDTA, pH 8.0) and checked for the expected PCR products.

### cDNA cloning and production of GST-human neutrophil arginase-1 fusion protein

The PCR products were directly cloned into the pCR 2.1 vector, using the TA cloning kit (Invitrogen) following the manufacturer's indications as previously described^47^. DNA sequencing was performed by ABI PRISM 3100-Avant Genetic Analyzer (Applied Biosystems, Carlsbad, California). DNA sequencing was performed on both strands from 5 independent cDNA clones. Full-length coding sequence for human neutrophil arginase-1 was amplified through PCR by using oligonucleotides flanked by EcoRI and XhoI cleavage sites and was subsequently subcloned into the bacterial expression vector pGEX-4T-1 (Pharmacia Biotech, Piscataway, NJ), obtaining the in-frame recombinant proteins composed of GST fused to the N terminus of recombinant neutrophil arginase-1. *Escherichia coli* BL-21 cells expressing GST or GST-human neutrophil arginase-1 fusion protein were grown in 400 ml of 2 × YT-G medium to A_600_ = 0.5–0.8, induced by the addition of 1 mM isopropyl-β-d-thiogalacto-pyranoside for 4 h. Cells were pelleted, resuspended in 20 ml PBS, and sonicated on ice by 4 pulses of 30 s each. Triton X-100 (1%, v/v) was added to the lysate and incubated for 30 min at 4 °C. Suspension was centrifuged at 12,000 rpm for 10 min in an SS34 rotor at 4 °C. The supernatant was mixed with 0.4 ml of a 50% slurry of glutathione-Sepharose 4B beads (Pharmacia Biotech) for 30 min at room temperature with gentle agitation as previously described^47^. Beads were sedimented and washed 3 times with PBS. Fusion protein or GST was eluted from the beads with 200 µl elution buffer (20 mM glutathione, 100 mM Tris–HCl, pH 8.0, 120 mM NaCl), analyzed by SDS–polyacrylamide gel electrophoresis and visualized by Coomassie Blue staining as previously described^47^.

### Cell transfection and RNA silencing

BxPC-3 cells (5 × 10^5^ cells) were transfected using Lipofectamine 2000 according to the manufacturer’s instructions (Invitrogen). ON-TARGETplus Human PERK (EIF2AK3) siRNA oligonucleotide mixtures, and scrambled siRNA (D-001810-01-05), used as control, were from Dharmacon. Transfection with siRNAs (50 nM) was performed using Lipofectamine 2000, according to the manufacturer’s instructions (Invitrogen).

### Western blot

Cells (5 × 10^6^) were lysed with 60 µl 25 mM Hepes (pH 7.7), 0.3 M NaCl, 1.5 mM MgCl_2_, 0.2 mM EDTA, 0.1% Triton X-100, 20 mM β-glycerophosphate, 0.1 mM sodium orthovanadate, supplemented with protease inhibitors (1 mM phenylmethylsulfonyl fluoride, 20 µg/ml aprotinin, 20 µg/ml leupeptin) as previously described^31^. Forty micrograms of proteins were run on SDS–polyacrylamide gels, transferred to nitrocellulose filters, blocked with 5% (w/v) nonfat dry milk in TBST (50 mM Tris–HCl, pH 8.0, 150 mM NaCl and 0.1% Tween 20) for 90 min at room temperature, and incubated for 1 h at room temperature or overnight at 4 °C with specific antibodies: mouse anti-27 kDa CHOP/GADD153 (Cell Signaling, Beverly, MA) (1:1000 dilution), anti-116 and 85 kDa PARP-1 (BD Pharmingen, Franklin Lakes, NJ) (1:500 dilution), anti-26 kDa, anti-Bap31 (Santa Cruz Biotechnology, Dallas, TX) (1:500 dilution), anti-42 kDa β-actin (Sigma-Aldrich) (1:5000 dilution); rabbit anti-38 kDa eIF2α (Cell Signaling) (1:1000 dilution), anti-38 kDa phospho-eIF2α (Cell Signaling) (1:1000 dilution), anti-37 kDa ATF4 (Abcam, Cambridge, UK) (1:500 dilution), anti-20 kDa cleaved caspase-3 (BD Pharmingen) (1:500 dilution), anti-20 kDa cleaved caspase-4 (Santa Cruz Biotechnology) (1:1000 dilution), anti-phospho PERK (Santa Cruz Biotechnology) (1:1000 dilution), anti-PERK (Santa Cruz Biotechnology) (1:1000 dilution), anti-20 kDa cleaved caspase-8 (Cell Signaling) (1:1000 dilution), anti-GRP78 Santa Cruz Biotechnology) (1:500 dilution). Anti-mouse (Amersham Biosciences, Buckinghamshire, UK) and anti-rabbit (Amersham Biosciences) IgG secondary HRP-antibodies were incubated at 1:5000 dilution in 5% (w/v) nonfat dry milk in TBST for 1 h at room temperature. Signals were developed using an enhanced chemiluminescence detection kit (Amersham Biosciences). The proteins of interest in Western blots were quantified by ImageJ software v1.53c (National Institutes of Health, MD; open source, https://imagej.nih.gov/ij/), and the gray values of protein bands from Western blots were normalized against its internal control β-actin, and expressed as values relative to the control. Detailed information on Western blot densitometry and quantification corresponding to the different gels is included in Supplementary Information_Blot densitometry.

### Statistical analysis

All statistical analyses in this study were performed using GraphPad Prism version 8.0.1 software (GraphPad, San Diego, CA, proprietary commercial software, https://www.graphpad.com/). Data analyses were conducted using one-way/two-way analysis of variance (ANOVA) when analyzing multiple sets of data, and the unpaired Student’s *t*-test to compare two data sets. The data are shown as mean ± standard deviation (SD). *P* < 0.05 was considered statistically significant.

## Supplementary Information

Supplementary Information.

## References

[1] Mollinedo F, Borregaard N, Boxer LA (1999). Novel trends in neutrophil structure, function and development. Immunol. Today.

[2] Mollinedo F (2006). Combinatorial SNARE Complexes modulate the secretion of cytoplasmic granules in human neutrophils. J. Immunol..

[3] Mollinedo F (2019). Neutrophil degranulation, plasticity, and cancer metastasis. Trends Immunol..

[4] Lecot P (2019). Neutrophil heterogeneity in cancer: From biology to therapies. Front. Immunol..

[5] Szczerba BM (2019). Neutrophils escort circulating tumour cells to enable cell cycle progression. Nature.

[6] Howard R, Kanetsky PA, Egan KM (2019). Exploring the prognostic value of the neutrophil-to-lymphocyte ratio in cancer. Sci. Rep..

[7] Ash DE (2004). Structure and function of arginases. J. Nutr..

[8] Munder M (2005). Arginase I is constitutively expressed in human granulocytes and participates in fungicidal activity. Blood.

[9] Munder M (2006). Suppression of T-cell functions by human granulocyte arginase. Blood.

[10] Wu G, Morris SM (1998). Arginine metabolism: Nitric oxide and beyond. Biochem. J..

[11] Canellakis ZN, Marsh LL, Bondy PK (1989). Polyamines and their derivatives as modulators in growth and differentiation. Yale J. Biol. Med..

[12] Pegg AE, Feith DJ (2007). Polyamines and neoplastic growth. Biochem. Soc. Trans..

[13] DeBerardinis RJ, Lum JJ, Hatzivassiliou G, Thompson CB (2008). The biology of cancer: Metabolic reprogramming fuels cell growth and proliferation. Cell Metab..

[14] Wheatley DN (2004). Controlling cancer by restricting arginine availability—Arginine-catabolizing enzymes as anticancer agents. Anticancer Drugs.

[15] Wheatley DN, Philip R, Campbell E (2003). Arginine deprivation and tumour cell death: Arginase and its inhibition. Mol. Cell Biochem..

[16] Wheatley DN (2005). Arginine deprivation and metabolomics: Important aspects of intermediary metabolism in relation to the differential sensitivity of normal and tumour cells. Semin. Cancer Biol..

[17] Riess C (2018). Arginine-depleting enzymes—An increasingly recognized treatment strategy for therapy-refractory malignancies. Cell Physiol. Biochem..

[18] Bobak YP, Vynnytska BO, Kurlishchuk YV, Sibirny AA, Stasyk OV (2010). Cancer cell sensitivity to arginine deprivation in vitro is not determined by endogenous levels of arginine metabolic enzymes. Cell Biol. Int..

[19] Cox JD (2001). Mechanistic and metabolic inferences from the binding of substrate analogues and products to arginase. Biochemistry.

[20] Mollinedo F, Lopez-Perez R, Gajate C (2008). Differential gene expression patterns coupled to commitment and acquisition of phenotypic hallmarks during neutrophil differentiation of human leukaemia HL-60 cells. Gene.

[21] Mollinedo F, Lazo PA (1997). Identification of two isoforms of the vesicle-membrane fusion protein SNAP-23 in human neutrophils and HL-60 cells. Biochem. Biophys. Res. Commun..

[22] Martin-Martin B, Nabokina SM, Lazo PA, Mollinedo F (1999). Co-expression of several human syntaxin genes in neutrophils and differentiating HL-60 cells: Variant isoforms and detection of syntaxin 1. J. Leukoc. Biol..

[23] Eckhart L (2001). Alternative splicing of caspase-8 mRNA during differentiation of human leukocytes. Biochem. Biophys. Res. Commun..

[24] Rotondo R (2011). Exocytosis of azurophil and arginase 1-containing granules by activated polymorphonuclear neutrophils is required to inhibit T lymphocyte proliferation. J. Leukoc. Biol..

[25] Hu H, Tian M, Ding C, Yu S (2019). The C/EBP homologous protein (CHOP) transcription factor functions in endoplasmic reticulum stress-induced apoptosis and microbial infection. Front. Immunol..

[26] Breckenridge DG, Stojanovic M, Marcellus RC, Shore GC (2003). Caspase cleavage product of BAP31 induces mitochondrial fission through endoplasmic reticulum calcium signals, enhancing cytochrome c release to the cytosol. J. Cell Biol..

[27] Pfaffenbach KT, Lee AS (2011). The critical role of GRP78 in physiologic and pathologic stress. Curr. Opin. Cell Biol..

[28] Danial NN, Korsmeyer SJ (2004). Cell death: Critical control points. Cell.

[29] Bressenot A (2009). Assessment of apoptosis by immunohistochemistry to active caspase-3, active caspase-7, or cleaved PARP in monolayer cells and spheroid and subcutaneous xenografts of human carcinoma. J. Histochem. Cytochem..

[30] Hitomi J (2004). Involvement of caspase-4 in endoplasmic reticulum stress-induced apoptosis and Abeta-induced cell death. J. Cell Biol..

[31] Gajate C (2012). Antitumor alkyl-lysophospholipid analog edelfosine induces apoptosis in pancreatic cancer by targeting endoplasmic reticulum. Oncogene.

[32] Nieto-Miguel T (2007). Endoplasmic reticulum stress in the proapoptotic action of edelfosine in solid tumor cells. Cancer Res..

[33] Bonilla X, el Dakir H, Mollinedo F, Gajate C (2015). Endoplasmic reticulum targeting in Ewing's sarcoma by the alkylphospholipid analog edelfosine. Oncotarget.

[34] Arumugam T (2009). Epithelial to mesenchymal transition contributes to drug resistance in pancreatic cancer. Cancer Res..

[35] Lee KM, Nguyen C, Ulrich AB, Pour PM, Ouellette MM (2003). Immortalization with telomerase of the Nestin-positive cells of the human pancreas. Biochem. Biophys. Res. Commun..

[36] Zhang Y, Lee C, Geng S, Li L (2019). Enhanced tumor immune surveillance through neutrophil reprogramming due to Tollip deficiency. JCI Insight.

[37] Jaillon S (2020). Neutrophil diversity and plasticity in tumour progression and therapy. Nat. Rev. Cancer.

[38] Wu L, Saxena S, Awaji M, Singh RK (2019). Tumor-associated neutrophils in cancer: Going pro. Cancers (Basel).

[39] Masucci MT, Minopoli M, Carriero MV (2019). Tumor associated neutrophils. Their role in tumorigenesis, metastasis, prognosis and therapy. Front. Oncol..

[40] Wang Y, Jonsson F (2019). Expression, role, and regulation of neutrophil fcgamma receptors. Front. Immunol..

[41] Matlung HL (2018). Neutrophils kill antibody-opsonized cancer cells by trogoptosis. Cell Rep..

[42] Lacal P, Pulido R, Sanchez-Madrid F, Mollinedo F (1988). Intracellular location of T200 and Mo1 glycoproteins in human neutrophils. J. Biol. Chem..

[43] Iurlaro R, Munoz-Pinedo C (2016). Cell death induced by endoplasmic reticulum stress. FEBS J..

[44] Jacobsen LC, Theilgaard-Monch K, Christensen EI, Borregaard N (2007). Arginase 1 is expressed in myelocytes/metamyelocytes and localized in gelatinase granules of human neutrophils. Blood.

[45] Mishalian I, Granot Z, Fridlender ZG (2017). The diversity of circulating neutrophils in cancer. Immunobiology.

[46] Rosales C (2018). Neutrophil: A cell with many roles in inflammation or several cell types?. Front. Physiol..

[47] Martin-Martin B, Nabokina SM, Blasi J, Lazo PA, Mollinedo F (2000). Involvement of SNAP-23 and syntaxin 6 in human neutrophil exocytosis. Blood.

[48] Mollinedo, F., Martin-Martin, B., Calafat, J., Nabokina, S. M. & Lazo, P. A. Role of vesicle-associated membrane protein-2, through Q-soluble *N*-ethylmaleimide-sensitive factor attachment protein receptor/R-soluble *N*-ethylmaleimide-sensitive factor attachment protein receptor interaction, in the exocytosis of specific and tertiary granules of human neutrophils. *J. Immunol.* **170**, 1034–1042 (2003). 10.4049/jimmunol.170.2.1034 12517971

[49] Herrero-Turrion MJ, Calafat J, Janssen H, Fukuda M, Mollinedo F (2008). Rab27a regulates exocytosis of tertiary and specific granules in human neutrophils. J. Immunol..

[50] Mollinedo, F. & Schneider, D. L. Subcellular localization of cytochrome *b* and ubiquinone in a tertiary granule of resting human neutrophils and evidence for a proton pump ATPase. *J. Biol. Chem.* **259**, 7143–7150 (1984). 6144682

[51] Mollinedo F, Manara FS, Schneider DL (1986). Acidification activity of human neutrophils. Tertiary granules as a site of ATP-dependent acidification. J. Biol. Chem..

[52] Mollinedo F, Schneider DL (1987). Intracellular organelle motility and membrane fusion processes in human neutrophils upon cell activation. FEBS Lett..

[53] Mollinedo F, Pulido R, Lacal PM, Sanchez-Madrid F (1991). Mobilization of gelatinase-rich granules as a regulatory mechanism of early functional responses in human neutrophils. Scand. J. Immunol..

[54] Lacal P, Pulido R, Sanchez-Madrid F, Cabanas C, Mollinedo F (1988). Intracellular localization of a leukocyte adhesion glycoprotein family in the tertiary granules of human neutrophils. Biochem. Biophys. Res. Commun..

[55] Quach A, Ferrante A (2017). The application of dextran sedimentation as an initial step in neutrophil purification promotes their stimulation, due to the presence of monocytes. J. Immunol. Res..

[56] Shaul ME, Fridlender ZG (2019). Tumour-associated neutrophils in patients with cancer. Nat. Rev. Clin. Oncol..

[57] Chu D, Dong X, Shi X, Zhang C, Wang Z (2018). Neutrophil-based drug delivery systems. Adv. Mater..

[58] Li Y (2020). Photosensitizer-laden neutrophils are controlled remotely for cancer immunotherapy. Cell Rep..

[59] Mollinedo F, Gajate C (2019). Novel therapeutic approaches for pancreatic cancer by combined targeting of RAF–>MEK–>ERK signaling and autophagy survival response. Ann. Transl. Med..

[60] Klimstra DS, Heffess CS, Oertel JE, Rosai J (1992). Acinar cell carcinoma of the pancreas. A clinicopathologic study of 28 cases. Am. J. Surg. Pathol..

[61] Skarda JS, Honick AB, Gibbins CS, Josselson AR, Rishi M (1994). Papillary-cystic tumor of the pancreas in a young woman: Fine-needle aspiration cytology, ultrastructure and DNA analysis. Diagn. Cytopathol..

[62] Mollinedo F (1997). Selective induction of apoptosis in cancer cells by the ether lipid ET-18-OCH_3_ (Edelfosine): Molecular structure requirements, cellular uptake, and protection by Bcl-2 and Bcl-X_L_. Cancer Res..

[63] Mollinedo F (2009). Novel anti-inflammatory action of edelfosine lacking toxicity with protective effect in experimental colitis. J. Pharmacol. Exp. Ther..

[64] Mollinedo F, Nieto JM, Andreu JM (1989). Cytoplasmic microtubules in human neutrophil degranulation: Reversible inhibition by the colchicine analogue 2-methoxy-5-(2',3',4'- trimethoxyphenyl)-2,4,6-cycloheptatrien-1- one. Mol. Pharmacol..

[65] David-Cordonnier MH (2005). DNA and non-DNA targets in the mechanism of action of the antitumor drug trabectedin. Chem. Biol..

[66] Santos-Beneit AM, Mollinedo F (2000). Expression of genes involved in initiation, regulation, and execution of apoptosis in human neutrophils and during neutrophil differentiation of HL-60 cells. J. Leukoc. Biol..

[67] Garcia-Navas R, Munder M, Mollinedo F (2012). Depletion of l-arginine induces autophagy as a cytoprotective response to endoplasmic reticulum stress in human T lymphocytes. Autophagy.

