# Supplementary material for: Neutrophils drive endoplasmic reticulum stress-mediated apoptosis in cancer cells through arginase-1 release
Source: Sci Rep. 2021 Jun 15;11:12574. doi: 10.1038/s41598-021-91947-0 (PMC8206108; doi:10.1038/s41598-021-91947-0)
Supplement: Supplementary file 1 — Supplementary Information. [file 41598_2021_91947_MOESM1_ESM.pdf]

SUPPLEMENTARY INFORMATION

Neutrophils drive endoplasmic reticulum stress-mediated apoptosis in cancer cells through arginase-1 release

Rósula García-Navas[1,2], Consuelo Gajate[1,3] and Faustino Mollinedo[1,3]

[1] *Instituto de Biología Molecular y Celular del Cáncer, Centro de Investigación del Cáncer, Consejo Superior de Investigaciones Científicas (CSIC)-Universidad de Salamanca, Campus Miguel de Unamuno, E-37007 Salamanca, Spain.*

[2] *Centro de Investigación Biomédica en Red de Cáncer (CIBERONC), Salamanca, Spain*

[3] *Laboratory of Cell Death and Cancer Therapy, Department of Molecular Biomedicine, Centro de Investigaciones Biológicas Margarita Salas, Consejo Superior de Investigaciones Científicas (CSIC), C/ Ramiro de Maeztu 9, E-28040 Madrid, Spain.*

Supplementary Figure S1

Supplementary Table S1

Western blot densitometry band quantification

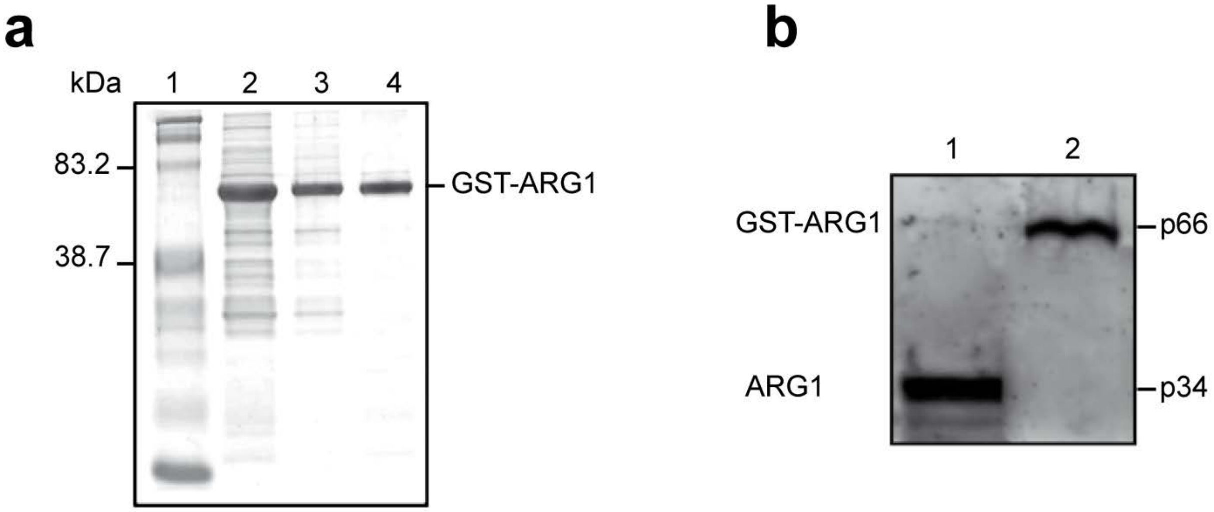

Supplementary Figure S1. GST-ARG1 overexpression in *E. coli*. (a) The gene corresponding to neutrophil arginase-1 (ARG1) was amplified from human neutrophils, and the gene product was cloned and overexpressed as GST-ARG1 in *E.coli*. The purification of the GST-ARG1 protein (66 kDa) was verified by SDS-PAGE. Lane 1: molecular weight markers; Lane 2: total protein extract; Lane 3: first purification with glutathione-Sepharose 4B beads; Lane 4: second purification step with glutathione-Sepharose 4B beads. (b) The polyclonal rabbit anti-ARG1 antibody recognizes both the native protein from neutrophil cell extracts (Lane 1, 30 µg total protein) and the recombinant protein GST-ARG1 (Lane 2, 5 µg of the second purification step). Molecular weights (in kilodaltons) are indicated at the right side of the panel.

Supplementary Table S1. IC_{80} values of GST-ARG1 on several human cancer cell lines. Data correspond to mean values of at least three independent experiments performed in triplicate. Standard deviation values were less than 10% in all cases.

**Cell line**	**Mean IC_{80} (mU/ml)**
COLO205	477.0
MCF7	163.5
SK-MEL-5	378.2
NCI-H522	469.0
OVCAR-3	493.6
PC-3	494.3
786-O	473.4
SF268	471.4
BxPC-3	84.9
HeLa	373.2

Western blot densitometry band quantification

Band intensity was quantified by ImageJ software v1.53c (National Institutes of Health, MD; Open source, https://imagej.nih.gov/ij/). For this purpose, developed films were scanned with Epson Perfection V700 (Epson Ibérica, S.A.U., Barcelona, Spain) using the EPSON Scan 3.9 software (Epson Ibérica, S.A.U, Barcelona, Spain). The films were scanned at 600 pixels per inch (ppi). The images were converted to 8-bit format to perform uncalibrated optical density (OD). After conversion, the background was subtracted using Rolling Ball Background Subtraction (ImageJ, https://imagej.net/Rolling_Ball_Background_Subtraction). Each band was individually selected with the rectangular ROI selection and “Gels” function, followed by quantification of peak area of the obtained histograms. Data were acquired as arbitrary area values. Arbitrary area values were normalized against its internal control β-actin or non-phosphorylated form, and expressed as values relative to the control.

The arbitrary area values for the corresponding Western blots in each figure are indicated below (data from three independent experiments for each experimental condition).

Figure 1

Figure 1a

HeLa		72 h	6 h	15 h	24 h	48 h	72 h
Caspase 3	p20	2219	8057	10823	7140	15078	25989
		1225	6281	11633	12241	12322	23298
		1920	8394	12989	10244	16843	27253
	p17	3949	7457	5422	7128	14006	25744
		8869	11393	12684	12838	13580	16220
		19488	15007	17118	19936	326	8086
PARP	p116	19961	20089	23558	12551	21324	2030
		25473	22861	19227	15748	13414	3840
		35365	31255	27344	10988	8139	5471
	p85	189	173	2952	3488	14675	18888
		229	784	1293	4982	12874	15788
		501	601	1959	6848	16969	19495
Actin	p42	12469	7902	9793	12942	14316	10750
		14496	15097	16040	10756	10754	13871
		17845	14913	12644	14820	15925	16114

Fold change vs Actin

HeLa		72 h	6 h	15 h	24 h	48 h	72 h
Caspase 3	p20	0.18	1.02	1.11	0.55	1.05	2.42
		0.08	0.42	0.73	1.14	1.15	1.68
		0.11	0.56	1.03	0.69	1.06	1.69
	p17	0.32	0.94	0.55	0.55	0.98	2.39
		0.61	0.75	0.79	1.19	1.26	1.17
		1.09	1.01	1.35	1.35	0.02	0.50
PARP	p116	1.60	2.54	2.41	0.97	1.49	0.19
		1.76	1.51	1.20	1.46	1.25	0.28
		1.98	2.10	2.16	0.74	0.51	0.34
	p85	0.02	0.02	0.30	0.27	1.03	1.76
		0.02	0.05	0.08	0.46	1.20	1.14
		0.03	0.04	0.15	0.46	1.07	1.21
Actin	p42	1.00	1.00	1.00	1.00	1.00	1.00
		1.00	1.00	1.00	1.00	1.00	1.00
		1.00	1.00	1.00	1.00	1.00	1.00

Figure 1b

**SF268**		72 h	6 h	15 h	24 h	48 h	72 h
Caspase 3	p20	3092	4613	2701	5119	12792	13686
		4489	4569	3859	10681	12945	14750
		7045	5414	6668	13459	11436	18017
	p17	2140	2034	3728	7105	14002	13374
		2069	4876	11626	5793	10667	16581
		2269	4402	4982	6408	15386	18316
PARP	p116	15350	15441	6242	7345	5201	2687
		11043	12660	8970	3021	1314	424
		11951	12779	4795	5006	2103	2004
	p85	824	1054	1987	2548	15364	20256
		1040	1594	1868	11374	15926	16644
		1677	1193	6872	13059	13839	19080
Actin	p42	12469	7902	9793	12942	14316	10750
		9029	9471	10372	10562	9347	9597
		9061	10288	11035	9627	8552	9951

Fold change vs Actin

**SF268**		72 h	6 h	15 h	24 h	48 h	72 h
Caspase 3	p20	0.25	0.58	0.28	0.40	0.89	1.27
		0.50	0.48	0.37	1.01	1.38	1.54
		0.78	0.53	0.60	1.40	1.34	1.81
	p17	0.17	0.26	0.38	0.55	0.98	1.24
		0.23	0.51	1.12	0.55	1.14	1.73
		0.25	0.43	0.45	0.67	1.80	1.84
PARP	p116	1.23	1.95	0.64	0.57	0.36	0.25
		1.22	1.34	0.86	0.29	0.14	0.04
		1.32	1.24	0.43	0.52	0.25	0.20
	p85	0.07	0.13	0.20	0.20	1.07	1.88
		0.12	0.17	0.18	1.08	1.70	1.73
		0.19	0.12	0.62	1.36	1.62	1.92
Actin	p42	1.00	1.00	1.00	1.00	1.00	1.00
		1.00	1.00	1.00	1.00	1.00	1.00
		1.00	1.00	1.00	1.00	1.00	1.00

Figure 3

Figure 3a

		fMLP	TNFα	PMA	37°C	4°C
ARG1	Spt	7120	814	830	444	808
		8333	976	620	998	626
		6811	662	398	175	336
	Pellet	9350	7824	8856	10780	7437
		10465	11599	10171	10936	95469
		15978	13459	14638	13944	15774
MPO	Spt	4568	324	276	579	166
		6207	531	321	637	189
		5631	999	543	740	135
	Pellet	9145	7696	5859	7366	9152
		7814	9584	8608	9663	8605
		8702	7397	8765	8934	7846
LF	Spt	3817	3471	4624	102	101
		6277	19495	13467	4066	3785
		9268	6065	6913	4866	15162
	Pellet	5498	5563	5392	4707	4147
		13983	10208	11268	2664	1746
		13972	7477	15780	12843	3862
MMP-9	Spt	4461	3338	4135	141	232
		11706	19476	18599	127	191
		9825	12055	13344	189	472
	Pellet	7877	8708	8236	7795	9452
		11949	12231	11877	13949	14779
		10228	9146	85551	9592	10509

Fold change Sn/Pellet

		fMLP	TNFα	PMA	37°C	4°C
ARG1	Spt	0.76	0.10	0.09	0.04	0.11
		0.80	0.08	0.06	0.09	0.01
		0.43	0.05	0.03	0.01	0.02
	Pellet	1.00	1.00	1.00	1.00	1.00
		1.00	1.00	1.00	1.00	1.00
		1.00	1.00	1.00	1.00	1.00
MPO	Spt	0.50	0.04	0.05	0.08	0.02

		0.79	0.06	0.04	0.07	0.02
		0.65	0.14	0.06	0.08	0.02
	Pellet	1.00	1.00	1.00	1.00	1.00
		1.00	1.00	1.00	1.00	1.00
		1.00	1.00	1.00	1.00	1.00
LF	Spt	0.69	0.62	0.86	0.02	0.02
		0.45	1.91	1.20	1.53	2.17
		0.66	0.81	0.44	0.38	3.93
	Pellet	1.00	1.00	1.00	1.00	1.00
		1.00	1.00	1.00	1.00	1.00
		1.00	1.00	1.00	1.00	1.00
MMP-9	Spt	0.57	0.38	0.50	0.02	0.02
		0.98	1.59	1.57	0.01	0.01
		0.96	1.32	0.16	0.02	0.04
	Pellet	1.00	1.00	1.00	1.00	1.00
		1.00	1.00	1.00	1.00	1.00
		1.00	1.00	1.00	1.00	1.00

Figure 4

Figure 4a

	Control	3 h	6 h	15 h	24 h	48 h
p-PERK	252	8765	17916	1701	1270	1003
	351	14532	11884	7509	1709	1159
	345	17442	10250	1537	7256	1107
PERK	13583	14290	12153	13188	10862	10511
	15397	19012	15663	15298	14802	16821
	17627	15094	12787	12702	12357	18548
p-eIF2a	100	5751	1392	11839	12322	9512
	583	4554	2769	18192	11416	7712
	859	4370	2091	11285	16709	5647
eIF2a	17566	16607	19322	14122	16732	18086
	17123	12703	14793	16554	16985	14445
	14110	13860	16365	17435	15321	15661
ATF4	771	7152	7747	8497	13064	14709
	673	1694	7721	10212	14929	11190
	701	1588	6438	11884	15519	17863
CHOP	450	1862	1558	9174	15722	16590
	1354	1617	1322	12614	19580	19325
	353	448	1785	11120	10534	11169
Caspase-8 (p20)	844	4667	5204	14414	10437	18292
	557	5589	11981	3253	10906	10176
	332	17429	2339	18372	8799	10240
Bap31 (p20)	276	403	1067	2140	5925	8460
	194	893	2169	2056	4797	7623
	355	1054	1868	9939	9731	10172
Caspase-4 (p20)	552	473	1218	7593	10846	11173
	589	372	1737	17586	11660	18991
	522	413	4718	7549	19408	13132
Caspase-3 (p20)	374	816	7235	15060	19735	14009
	468	571	2374	12621	14771	18518
	655	794	8033	12786	19225	19626
PARP-1 (p85)	604	1193	7631	13896	16764	14286
	532	1404	7106	13811	12468	9246
	792	1913	8886	12510	13501	8382
GRP78	13641	13231	15453	15181	13320	12776
	15977	15474	7461	4517	12994	12227
	12072	16886	14436	18119	17290	15141

Actin	15722	14534	15260	16084	19163	16789
	13289	11546	13571	9655	13861	9266
	10800	10543	10181	10382	9913	9812

Fold change

	Control	3 h	6 h	15 h	24 h	48 h
p-PERK (p-PERK/PERK)	0.02	0.61	1.47	0.13	0.12	0.10
	0.02	0.76	0.76	0.49	0.12	0.07
	0.02	1.16	0.80	0.12	0.59	0.06
PERK (PERK/Actin)	0.86	0.98	0.80	0.82	0.57	0.63
	1.16	1.65	1.15	1.58	1.07	1.82
	1.63	1.43	1.26	1.22	1.25	1.89
p-eIF2a (p-eIF2a/eIF2a)	0.01	0.35	0.07	0.84	0.74	0.53
	0.03	0.36	0.19	1.10	0.67	0.53
	0.06	0.32	0.13	0.65	1.09	0.36
eIF2a (eIF2a/Actin)	1.12	1.14	1.27	0.88	0.87	1.08
	1.29	1.10	1.09	1.71	1.23	1.56
	1.31	1.31	1.61	1.68	1.55	1.60
ATF4 (ATF4/Actin)	0.05	0.49	0.51	0.53	0.68	0.88
	0.05	0.15	0.57	1.06	1.08	1.21
	0.06	0.15	0.63	1.14	1.57	1.82
CHOP (CHOP/Actin)	0.03	0.13	0.10	0.57	0.82	0.99
	0.10	0.14	0.10	1.31	1.41	2.09
	0.03	0.04	0.18	1.07	1.06	1.14
Caspase-8 (p20) (C8/Actin)	0.05	0.32	0.34	0.90	0.54	1.09
	0.04	0.48	0.88	0.34	0.79	1.10
	0.03	1.65	0.23	1.77	0.89	1.04
Bap31 (p20) (Bap31/Actin)	0.02	0.03	0.07	0.13	0.31	0.50
	0.01	0.08	0.16	0.21	0.35	0.82
	0.03	0.10	0.18	0.96	0.98	1.04
Caspase-4 (p20) (C4/Actin)	0.04	0.03	0.08	0.47	0.57	0.67
	0.04	0.03	0.13	1.82	0.84	2.05
	0.05	0.04	0.46	0.73	1.96	1.34
Caspase-3 (p20) (C3/Actin)	0.02	0.06	0.47	0.94	1.03	0.83
	0.04	0.05	0.17	1.31	1.07	2.00
	0.06	0.08	0.79	1.23	1.94	2.00
PARP-1 (p85) (PARP-1/Actin)	0.04	0.08	0.50	0.86	0.87	0.85
	0.04	0.12	0.52	1.43	0.90	1.00
	0.07	0.18	0.87	1.20	1.36	0.85

GRP78 (GRP78/Actin)	0.87	0.91	1.01	0.94	0.70	0.76
	1.20	1.34	0.55	0.47	0.94	1.32
	1.12	1.60	1.42	1.75	1.74	1.54
Actin	1.00	1.00	1.00	1.00	1.00	1.00
	1.00	1.00	1.00	1.00	1.00	1.00
	1.00	1.00	1.00	1.00	1.00	1.00

Figure 4b

	Control	PMN-Spt	z-LEVD+PMN-Spt
Caspase-4 (p20)	183	6750	266
	158	9485	593
	418	5403	260
Actin	13798	12402	13632
	16577	13242	12106
	15662	14341	14363

Fold change

	Control	PMN-Spt	z-LEVD+PMN-Spt
Caspase-4 (p20) (C4/Actin)	0.01	0.54	0.02
	0.01	0.72	0.05
	0.03	0.38	0.02
Actin	1.00	1.00	1.00
	1.00	1.00	1.00
	1.00	1.00	1.00

Figure 4c

	Control	PMN-Spt	z-IETD+PMN-Spt
Caspase-8 (p20)	317	15686	171
	100	15925	128
	388	14211	473
Bap-31 (p20)	463	4714	614
	448	9856	537
	673	5601	989
Actin	12501	11539	12861
	15270	16132	17675
	15459	14482	15502

Fold change

	Control	PMN-Spt	z-IETD+PMN-Spt
Caspase-8 (p20) (C8/Actin)	0.03	1.36	0.01
	0.01	0.99	0.01
	0.03	0.98	0.03
Bap31 (p20) (Bap31/Actin)	0.04	0.41	0.05
	0.03	0.61	0.03
	0.04	0.39	0.06

Figure 5

Figure 5a

		Control	3 h	6 h	15 h
Caspase-3 (p20)		953	2050	9741	9519
		942	2271	9192	7862
		599	1083	7474	9557
PARP-1 (p85)		600	975	8655	8935
		721	531	5427	7707
		552	869	9971	9233
Actin		14786	14366	14624	13587
		13594	14626	13447	13582
		13063	14259	13722	14332

Fold change

	Control	3 h	6 h	15 h
Caspase-3 (p20) (C3/Actin)	0.06	0.14	0.66	0.64
	0.07	0.17	0.68	0.58
	0.05	0.08	0.57	0.73
PARP-1 (p85) (PARP-1/Actin)	0.04	0.07	0.59	0.60
	0.05	0.04	0.40	0.57
	0.04	0.07	0.76	0.71
Actin	1.00	0.97	0.99	0.92
	1.00	1.08	0.99	1.00
	1.00	1.09	1.05	1.10

Figure 5b

	Control	3 h	6 h	15 h	24 h	48 h
p-PERK	203	12770	8630	583	542	519
	388	14161	9165	465	478	489
	279	11422	7485	357	693	534
PERK	12473	10480	13689	10874	12196	12409
	14391	10250	12105	14742	10391	11438
	14193	10125	13259	12636	14095	11719
p-eIF2a	1439	2749	11878	12793	11400	7290
	1390	4895	12934	10289	11433	7369
	1006	2565	13822	13366	11457	6761
eIF2a	13669	11941	14652	12131	13090	14363
	14534	12879	12175	10871	14634	10642

	10863	12586	11415	10723	13626	11372
ATF4	1675	1136	3595	11064	1361	2331
	1470	1308	6640	12224	1417	2415
	1954	1791	4378	6385	1170	1603
CHOP	1735	1085	8678	9407	8425	1259
	1978	1830	9192	9953	7777	2555
	1887	773	7384	8664	8825	2966
Caspase-8 (p20)	223	305	1849	11293	6275	9481
	242	271	1081	11787	6243	12102
	288	417	1648	9268	10528	11395
Bap31 (p20)	160	1540	3002	10694	7925	9755
	165	1223	4551	12213	10950	9764
	230	1264	5339	8741	6062	6131
Caspase-4 (p20)	283	2579	5858	12145	9274	9190
	212	1202	7217	11521	12109	7929
	330	2682	6645	6806	6125	8673
GRP78	11244	11137	11347	11164	13305	12195
	11207	12388	13659	11308	11375	12556
	11007	12339	12303	11762	11240	12935
Actin	13265	12821	13136	13354	13081	12734
	12884	13344	13996	13577	12630	11983
	12352	13821	12623	13989	11511	11897

Fold Change

	Control	3 h	6 h	15 h	24 h	48 h
p-PERK (p-PERK/PERK)	0.02	1.22	0.63	0.05	0.04	0.04
	0.03	1.38	0.76	0.03	0.05	0.04
	0.02	1.13	0.56	0.03	0.05	0.05
PERK (PERK/Actin)	0.94	0.82	1.04	0.81	0.93	0.97
	1.12	0.77	0.86	1.09	0.82	0.95
	1.15	0.73	1.05	0.90	1.22	0.99
p-eIF2a (p-eIF2a/eIF2a)	0.11	0.23	0.81	1.05	0.87	0.51
	0.10	0.38	1.06	0.95	0.78	0.69
	0.09	0.20	1.21	1.25	0.84	0.59
eIF2a (eIF2a/Actin)	1.03	0.93	1.12	0.91	1.00	1.13
	1.13	0.97	0.87	0.80	1.16	0.89
	0.88	0.91	0.90	0.77	1.18	0.96
ATF4 (ATF4/Actin)	0.13	0.09	0.27	0.83	0.10	0.18
	0.11	0.10	0.47	0.90	0.11	0.20

	0.16	0.13	0.35	0.46	0.10	0.13
CHOP (CHOP/Actin)	0.13	0.08	0.66	0.70	0.64	0.10
	0.15	0.14	0.66	0.73	0.62	0.21
	0.15	0.06	0.58	0.62	0.77	0.25
Caspase-8 (p20) (C8/Actin)	0.02	0.02	0.14	0.85	0.48	0.74
	0.02	0.02	0.08	0.87	0.49	1.01
	0.02	0.03	0.13	0.66	0.91	0.96
Bap31 (p20) (Bap31/Actin)	0.01	0.12	0.23	0.80	0.61	0.77
	0.01	0.09	0.33	0.90	0.87	0.81
	0.02	0.09	0.42	0.62	0.53	0.52
Caspase-4 (p20) (C4/Actin)	0.02	0.20	0.45	0.91	0.71	0.72
	0.02	0.09	0.52	0.85	0.96	0.66
	0.03	0.19	0.53	0.49	0.53	0.73
GRP78 (GRP78/Actin)	0.85	0.87	0.86	0.84	1.02	0.96
	0.87	0.93	0.98	0.83	0.90	1.05
	0.89	0.89	0.97	0.84	0.98	1.09
Actin	1.00	1.00	1.00	1.00	1.00	1.00
	1.00	1.00	1.00	1.00	1.00	1.00
	1.00	1.00	1.00	1.00	1.00	1.00

Figure 5c

	Control	PMN-Spt	z-LEVD+PMN-Spt
Caspase-4 (p20)	646	6966	2587
	417	6744	2003
	867	7053	1726
Actin	11287	11117	14203
	14213	12317	11480
	11178	12893	13674

Fold Change

	Control	PMN-Spt	z-LEVD+PMN-Spt
Caspase-4 (p20) (C4/Actin)	0.06	0.63	0.18
	0.03	0.55	0.17
	0.08	0.55	0.13
Actin	1.00	1.00	1.00
	1.00	1.00	1.00
	1.00	1.00	1.00

Figure 6

Figure 6a

	Control	fMLP	nor-NOHA
p-PERK	202	8998	240
	383	10033	407
	372	7965	364
PERK	6226	8996	7850
	9830	10124	5625
	8763	6303	7636
p-eIF2a	197	5355	272
	330	8103	430
	165	5332	242
eIF2a	10659	9656	9120
	5511	8730	10364
	8536	5558	9270
ATF4	208	6206	367
	378	7326	432
	330	9180	425
CHOP	170	9278	373
	328	10998	191
	195	6692	196
Caspase-8 (p20)	340	5267	515
	298	9690	222
	329	5142	416
Bap31 (p20)	389	5085	449
	243	8749	268
	121	6593	295
Caspase-4 (p20)	276	8429	492
	319	7139	448
	182	8997	386
Caspase-3 (p20)	335	10365	8978
	229	7275	8449
	157	10299	5710
PARP-1 (p86)	393	9799	5460
	353	8310	6185
	298	5094	10930
Actin	12392	11151	9386
	13234	11878	14629
	11480	11496	13696

Fold change

	Control	fMLP	nor-NOHA
p-PERK (p-PERK/PERK)	0.03	1.00	0.03
	0.04	0.99	0.07
	0.04	1.26	0.05
PERK (PERK/Actin)	0.50	0.81	0.84
	0.74	0.85	0.38
	0.76	0.55	0.56
p-eIF2a (p-eIF2a/eIF2a)	0.02	0.55	0.03
	0.06	0.93	0.04
	0.02	0.96	0.03
eIF2a (eIF2a/Actin)	0.86	0.87	0.97
	0.42	0.73	0.71
	0.74	0.48	0.68
ATF4 (ATF4/Actin)	0.02	0.56	0.04
	0.03	0.62	0.03
	0.03	0.80	0.03
CHOP (CHOP/Actin)	0.01	0.83	0.04
	0.02	0.93	0.01
	0.02	0.58	0.01
Caspase-8 (p20) (C8/Actin)	0.03	0.47	0.05
	0.02	0.82	0.02
	0.03	0.45	0.03
Bap31 (p20) (Bap31/Actin)	0.03	0.46	0.05
	0.02	0.74	0.02
	0.01	0.57	0.02
Caspase-4 (p20) (C4/Actin)	0.02	0.76	0.05
	0.02	0.60	0.03
	0.02	0.78	0.03
Caspase-3 (p20) (C3/Actin)	0.03	0.93	0.96
	0.02	0.61	0.58
	0.01	0.90	0.42
PARP-1 (p85) (PARP-1/Actin)	0.03	0.88	0.58
	0.03	0.70	0.42
	0.03	0.44	0.80
Actin	1.00	1.00	1.00
	1.00	1.00	1.00
	1.00	1.00	1.00

Figure 6b

	siRNA Control	siRNA PERK
PERK	11113	667
	12531	752
	10742	645
Actin	10655	11263
	8470	9207
	9140	12530

Fold change

	siRNA Control	siRNA PERK
PERK (PERK/Actin)	1.04	0.06
	1.48	0.08
	1.18	0.05
Actin	1.00	1.00
	1.00	1.00
	1.00	1.00

Figure 6c

	siRNA control	siRNA control + PMN-Spt	siRNA PERK + PMN-Spt
p-eIF2a	348	11037	2255
	184	7931	3250
	312	6861	2967
eIF2a	12241	12259	9871
	10457	6735	12835
	14687	5927	14387
ATF4	248	10334	4038
	273	11307	4595
	410	8156	5117
CHOP	198	12129	4886
	378	5288	1070
	111	7854	1350
Caspase-8 (p20)	183	8733	1646
	336	10683	1966
	112	10466	1599
Bap31 (p20)	245	8433	1180

	232	5123	1325
	217	5638	1952
Caspase-4 (p20)	270	11848	9103
	251	5980	10310
	415	10492	5717
Caspase-3 (p20)	293	7166	10241
	416	11722	11264
	391	6752	7906
PARP-1 (p85)	272	8760	9827
	126	10579	10181
	139	8559	11146
Actin	8631	10511	10549
	9905	12556	10076
	12291	9124	12054

Fold change	siRNA control	siRNA control + PMN-Spt	siRNA PERK + PMN-Spt
p-eIF2a (p-eIF2a/eIF2a)	0.03	0.90	0.23
	0.02	1.18	0.25
	0.02	1.16	0.21
eIF2a (eIF2a/Actin)	1.42	1.17	0.94
	1.06	0.54	1.27
	1.19	0.65	1.19
ATF4 (ATF4/Actin)	0.03	0.98	0.38
	0.03	0.90	0.46
	0.03	0.89	0.42
CHOP (CHOP/Actin)	0.02	1.15	0.46
	0.04	0.42	0.11
	0.01	0.86	0.11
Caspase-8 (p20) (C8/Actin)	0.02	0.83	0.16
	0.03	0.85	0.20
	0.01	1.15	0.13
Bap31 (p20) (Bap31/Actin)	0.03	0.80	0.11
	0.02	0.41	0.13
	0.02	0.62	0.16
Caspase-4 (p20)	0.03	1.13	0.86
	0.03	0.48	1.02

(C4/Actin)	0.03	1.15	0.47
Caspase-3 (p20) (C3/Actin)	0.03	0.68	0.97
	0.04	0.93	1.12
	0.03	0.74	0.66
PARP-1 (p85) (PARP-1/Actin)	0.03	0.83	0.93
	0.01	0.84	1.01
	0.01	0.94	0.92
Actin	1.00	1.00	1.00
	1.00	1.00	1.00
	1.00	1.00	1.00
